# Protective role of aqueous *Coriandrum sativum* seed extract in diet-induced glucolipid metabolic disorder through gut–liver axis regulation

**DOI:** 10.3389/fendo.2026.1744741

**Published:** 2026-02-13

**Authors:** Yuru Xu, Ying Cao, Linen Zou, Wulanna, Xinyu Liu, Shutong Yan, Chengyi Liu, Mengran Gao, Jingke Zhan, Qingchun Wang, Chongming Wu

**Affiliations:** 1 School of Chinese Materia Medica, Tianjin University of Traditional Chinese Medicine, Tianjin, China; 2 Medical Administration Section, Hohhot Hospital of Mongolian and Chinese Medicine, Hohhot, China; 3 Inner Mongolia Academy of Traditional Chinese and Mongolian Medicine, Hohhot, China; 4 Tianjin Key Laboratory of Therapeutic Substance of Traditional Chinese Medicine, Tianjin, China; 5 State Key Laboratory of Chinese Medicine Modernization, Tianjin, China

**Keywords:** *Coriandrum sativum* seed, glucose metabolism disorders, gut microbiome, lipid metabolism disorders, oxidative stress

## Abstract

**Objective:**

To elucidate the protective effects of aqueous *Coriandrum sativum* seed extract against high-fat, high-sugar diet (HSFD)-induced glucolipid metabolic disorder in mice, with particular focus on gut–liver axis regulation involving hepatic metabolism, oxidative stress, inflammation, and gut microbiota composition.

**Methods:**

Male mice were fed an HSFD and orally treated with *Coriandrum sativum* seed extract (1.0 or 2.0 g/kg/day) for eight weeks. Biochemical parameters, histopathology, hepatic gene expression, oxidative stress markers, and gut microbial profiles were assessed via standard assays, RT-qPCR, Western blot, histological staining, and full-length 16S rRNA gene sequencing with functional prediction.

**Results:**

The extract significantly ameliorated HSFD-induced metabolic impairments, including hyperglycemia, hyperlipidemia, insulin resistance, and hepatic steatosis. Histological improvements were observed in the liver, pancreas, and colon. Hepatic expression of FAS, NF-κB, and IL-6 was suppressed, while PPARα and LDLR expression was restored. Antioxidant defenses were enhanced by reducing malondialdehyde and increasing superoxide dismutase activity. Microbiota analysis revealed partial restoration of beneficial taxa such as *Lactobacillus murinus* and *Lachnospiraceae*_UCG-006, alongside enrichment of microbial pathways related to energy and carbohydrate metabolism.

**Conclusion:**

Aqueous *Coriandrum sativum* seed extract exerts systemic metabolic benefits in diet-induced glucolipid dysregulation by targeting the gut–liver axis. Its multi-targeted actions on hepatic metabolism, inflammatory signaling, oxidative balance, and gut microbiota composition support its potential as a natural therapeutic agent for metabolic disorders.

## 1 Introduction

Metabolic disorders, such as obesity, insulin resistance, and dyslipidemia, have emerged as a significant global health concern, contributing to the rising incidence of chronic diseases like type 2 diabetes, cardiovascular diseases, and non-alcoholic fatty liver disease (1–3). These disorders are primarily caused by poor dietary patterns, particularly the excessive consumption of high-fat and high-sugar diets (HSFD), which disrupt lipid metabolism, glucose homeostasis, and liver function, leading to the development of metabolic syndrome (4, 5). Metabolic syndrome is a complex condition that includes visceral fat accumulation, insulin resistance, dyslipidemia, and chronic inflammation, and it is considered a precursor to a range of life-threatening diseases (6, 7). Specifically, visceral adipose tissue serves not only as an energy storage organ but also as an active endocrine organ. It secretes various adipokines and pro-inflammatory cytokines, such as tumor necrosis factor-α and interleukin-6, which can exacerbate systemic low-grade inflammation and insulin resistance (8, 9). Insulin resistance further leads to compensatory hyperinsulinemia, promoting increased hepatic synthesis of very-low-density lipoprotein and hypertriglyceridemia, accompanied by reduced high-density lipoprotein cholesterol, thereby contributing to the formation of typical atherogenic dyslipidemia (10). Moreover, persistent energy surplus and metabolic abnormalities can induce mitochondrial dysfunction, generating reactive oxygen species and triggering oxidative stress. Oxidative stress not only directly damages cellular structures but also activates inflammatory signaling pathways such as NF-κB, establishing a vicious cycle between inflammation and oxidative stress that accelerates pancreatic β-cell dysfunction, vascular endothelial impairment, and the progression of non-alcoholic fatty liver disease (11–14).

Among various metabolic diseases, type 2 diabetes stands out prominently, as its pathogenesis extends far beyond insulin deficiency or insulin resistance. Recent research indicates that the development and progression of diabetes are closely linked to the interplay of dysfunctions across multiple organs and systems. For instance, gut microbiome dysbiosis may contribute to insulin resistance by affecting short-chain fatty acid production, intestinal barrier function, and systemic inflammation (15). A global study revealed that specific foodborne pathogens, such as *Staphylococcus aureus*, might be associated with the risk of diabetes, highlighting the potential role of environmental microbial exposure in metabolic disorders (1). In the pathological progression of diabetic complications, mitochondrial dysfunction occupies a central position. Particularly in the development of diabetic cardiomyopathy, signaling pathways such as Adipsin and Irak2 have been identified as key molecular targets regulating cardiomyocyte energy metabolism homeostasis and apoptotic processes (2). Furthermore, the functional decline of pancreatic β-cells is a critical factor in the progression of type 2 diabetes. Single-cell sequencing technology enables precise quantification of “risk cells” in pancreatic islets and their dynamic changes, offering novel insights into the heterogeneity and adaptive responses of islet cells during diabetes progression (3). These research advances underscore the complexity of diabetes as a systemic, multifactorial disease.

While conventional pharmacological therapies—such as insulin-sensitizing agents, lipid-lowering drugs, and anti-inflammatory medications—are commonly employed to manage metabolic diseases, long-term use of these drugs often leads to adverse effects, including gastrointestinal discomfort, muscle pain, and liver toxicity (16, 17). Consequently, there is increasing interest in the use of natural products, particularly herbal medicines, as adjunctive or alternative therapies for managing metabolic diseases (18–20). Traditional Chinese herbal medicine and the concept of “medicinal food homology” emphasize the synergistic effects of multiple components derived from natural foods and herbs to holistically regulate the body’s balance. This multi-targeted therapeutic approach simultaneously addresses key pathways such as lipid metabolism, glucose regulation, inflammation, and gut microbiota modulation (21). Numerous studies have confirmed that many natural bioactive components, such as flavonoids and phenolic acids, can improve insulin sensitivity, suppress inflammation, and alleviate oxidative stress by modulating key signaling pathways including AMPK/PI3K/Akt and Nrf2-ARE, thereby exerting therapeutic effects against metabolic syndrome (22, 23).This approach is particularly attractive for individuals seeking safer, more holistic treatment options (24, 25).

One such herbal remedy is *Coriandrum sativum*, a plant traditionally used in culinary and medicinal practices across many cultures (26–28). While much of the existing research on *Coriandrum sativum* has focused on its leaves and essential oils (28–30), *Coriandrum sativum* seeds remain underexplored, especially in the context of metabolic health. The seeds of *Coriandrum sativum* contain a variety of bioactive compounds, including flavonoids, phenolic acids, and essential oils, all of which have been shown to possess antioxidant, anti-inflammatory, and antidiabetic properties (27, 31, 32). Several studies have suggested that *Coriandrum sativum* seed extract has lipid-lowering and blood glucose-regulating effects (28, 33), making it a promising candidate for the management of metabolic disorders (34). However, the mechanisms by which *Coriandrum sativum* seed extract exerts these effects—particularly in the context of diet-induced metabolic dysfunction—remain poorly understood (28).

Gut microbiota has gained increasing attention as a crucial regulator of metabolic health. The gut microbiota plays an essential role in regulating nutrient absorption, fat storage, and glucose metabolism (26, 35–37). Disruptions to the gut microbiota, known as dysbiosis, have been linked to the development of obesity, insulin resistance, and NAFLD (38, 39). Dysbiosis results in intestinal inflammation, increased gut permeability, and systemic inflammation, all of which contribute to the pathophysiology of metabolic diseases (40). Dietary interventions, including the use of herbal extracts, have been shown to positively modulate gut microbiota composition, restoring microbial balance and improving metabolic health (41, 42). However, the impact of *Coriandrum sativum* seed extract on gut microbiota composition in the context of HSFD-induced metabolic disorders has not been adequately explored.

In addition to its effects on the gut microbiota, the regulation of gene expression involved in lipid metabolism and inflammation is crucial for maintaining metabolic homeostasis. Key genes such as *PPARα*, *FAS*, *LDLR*, *NF-κB*, and *IL-6* are central to the regulation of these metabolic pathways (43, 44). Dysregulation of these genes is commonly observed in individuals with metabolic disorders. Although previous studies have demonstrated that herbal extracts can influence the expression of these genes (45–47), the specific effects of *Coriandrum sativum* seed extract on the regulation of these genes, particularly in the context of HSFD-induced metabolic dysfunction, remain underexplored. Understanding how *Coriandrum sativum* seed extract modulates these pathways could offer valuable insights into its potential as a multi-targeted therapeutic for managing metabolic diseases.

This study aims to explore the therapeutic potential of *Coriandrum sativum* seed aqueous extract in the context of HSFD-induced metabolic disorders. Specifically, this research will evaluate the effects of *Coriandrum sativum* seed extract on lipid metabolism, glucose regulation, gut microbiota composition, and gene expression related to lipid metabolism and inflammation. The primary goal is to examine whether *Coriandrum sativum* seed extract can act as an effective multi-targeted intervention to restore metabolic balance, providing a natural and holistic approach to the management of metabolic disorders. By investigating these mechanisms, the study will offer new insights into the role of *Coriandrum sativum* seed extract in improving metabolic health, providing evidence for its potential use as a complementary therapy in the treatment of metabolic diseases.

## 2 Materials and methods

### 2.1 GC-MS analysis of aqueous *Coriandrum sativum* seed extract

Dried *Coriandrum sativum* seeds were purchased from TongRenTang Chinese Medicine (Beijing, China). A total of 100 g of coarsely ground seeds was extracted twice with boiling water. The first extraction was performed with 1000 mL of water for 1 hour, followed by filtration. The residue was then re-extracted with 800 mL of water for 45 minutes. Both filtrates were combined and concentrated under reduced pressure to a final volume of 100 mL, and stored at 4 °C.

For sample preparation, 1 mL of the concentrated extract was mixed with 1 mL of ethyl acetate and vortexed for 60 seconds using an XW-80A vortex mixer (QiLinBeiEr, China). After centrifugation at 12,000 rpm for 5 minutes at 4 °C (H1750R centrifuge, XiangYi, China), the upper organic layer was filtered through a 0.22 μm membrane and evaporated to dryness under nitrogen using an MD200–1 nitrogen evaporator (AoSheng, China). The residue was reconstituted in 200 μL ethyl acetate and stored at −20 °C for GC-MS analysis.

GC-MS analysis was carried out using a GCMS-QP2010SE instrument (Shimadzu, Japan) equipped with an EI ion source and a DB-5MS capillary column (30 m × 0.25 mm, 0.25 μm). The injection volume was 1 μL in splitless mode. The injector temperature was set at 250 °C. The column oven temperature was programmed as follows: 70 °C (held for 2 min), increased to 180 °C at 2 °C/min (held for 5 min), then to 230 °C at 10 °C/min (held for 5 min). The total run time was 72 minutes. Helium was used as the carrier gas at a constant flow of 1.0 mL/min.

Mass spectrometric detection was performed in full-scan mode (m/z 40–350) with the following settings: EI energy at 70 eV, ion source temperature 200 °C, interface temperature 230 °C, quadrupole temperature 150 °C, scan speed 1111 amu/s, and a solvent delay of 6 minutes.

Compounds were identified by comparing their mass spectra with entries in the NIST 17 library, with only those showing a match factor ≥90% being retained. When multiple matches were available, the compound with the highest similarity was selected. Relative abundances were calculated using peak area normalization.

### 2.2 Animals and experimental design

A total of thirty-two male KM mice (5 weeks old, 32–34 g) were procured from SPF (Beijing) Biotechnology Co., Ltd. (Beijing, China). Animals were housed under specific pathogen-free (SPF) conditions at Tianjin University of Traditional Chinese Medicine, maintained at a controlled temperature (24 ± 1 °C), relative humidity of 50%–70%, and a 12 h light/dark cycle. All experimental procedures were conducted in accordance with the ARRIVE guidelines and approved by the Animal Experiment Ethics Commit-tee of Tianjin University of Traditional Chinese Medicine (Approval No. TCM-LAEC2024162z1030).

Following acclimatization, mice were randomly assigned to four groups (n = 7 per group): Normal Diet (ND), High-Sugar High-Fat Diet (HSFD), low-dose aqueous *Coriandrum sativum* seed extract (YS-L, 1.0 g/kg), and high-dose aqueous *Coriandrum sativum* seed extract (YS-H, 2.0 g/kg). Mice in the ND group were provided with standard chow and distilled water, while those in the HSFD, YS-L, and YS-H groups received a high-sugar high-fat diet comprising 67% standard chow, 10% lard, 20% sucrose, 2.5% cholesterol, and 0.5% sodium cholate, along with 20% fructose solution ad libitum (SPF Bio-technology Co., Ltd., Beijing, China). In addition, mice in the YS-L and YS-H groups were administered low- and high-dose YS aqueous extract via oral gavage daily for 56 consecutive days; control groups (ND and HSFD) received an equivalent volume of saline.

Upon completion of the intervention, all mice were anesthetized with an intraperitoneal injection of pentobarbital sodium at a dosage of 50 mg/kg. Blood samples were collected via retro-orbital bleeding, after which the animals were euthanized by cervical dislocation. Following cardiac perfusion with phosphate-buffered saline (PBS, Beijing Solarbio Science & Technology Co., Ltd., Beijing, China), liver, pancreas, and colon tissues were harvested. Portions of each tissue were fixed in 4% paraformaldehyde for histological analysis, while remaining samples were snap-frozen in liquid nitrogen and stored at −80 °C for subsequent biochemical assays. Additionally, cecal contents were collected and stored at −80 °C for subsequent microbial genomic analysis via full-length 16S rRNA sequencing.

### 2.3 Assessment of biochemical parameters in serum and liver tissues

On day 56 of the intervention period, fasting blood glucose (FBG) was measured using tail tip blood samples collected with a calibrated glucometer (ACCU-CHEK Instant S, Roche Diagnostics, Germany). For serum preparation, whole blood was obtained via retro-orbital venous plexus puncture. Samples were allowed to clot at room temperature for 2 hours, then centrifuged at 4500 rpm for 10 minutes at 4 °C. The resulting serum was collected for lipid profiling. Serum levels of total cholesterol (TC, Cat# A111-1-1), triglycerides (TG, Cat# A110-1-1), low-density lipoprotein cholesterol (LDL-C, Cat# A113-1-1), and high-density lipoprotein cholesterol (HDL-C, Cat# A112-1-1) were determined using commercial enzymatic colorimetric assay kits (Nan-jing Jiancheng Bioengineering Institute, Nanjing, China) according to the manufacturer’s instructions.

Liver tissues were homogenized in ice-cold saline (1:9, w/v), and the homogenates were centrifuged at 2000 rpm for 10 minutes at 4°C. The resulting supernatants were used for quantification of hepatic biochemical indices, including TC, TG, alkaline phosphatase (AKP, Cat# A059-2-1), alanine aminotransferase (ALT, Cat# C009-2-1), aspartate aminotransferase (AST, Cat# C010-2-1), superoxide dismutase (SOD, Cat# A001-1-1), malondialdehyde (MDA, Cat# A003-1-1), and glycogen (Cat# A043-1-1), using corresponding assay kits (Nanjing Jiancheng Bioengineering Institute).

Serum insulin concentrations were determined using a mouse insulin ELISA kit (Jiangsu Meimian Industrial Co., Ltd., Jiangsu, China) following the manufacturer’s instructions. Briefly, serum samples were added to 96-well microplates pre-coated with purified mouse insulin antibodies and incubated with horseradish peroxidase (HRP)-conjugated detection antibodies. After thorough washing to remove unbound components, chromogenic substrate solution was applied, and the colorimetric reaction was carried out at 37°C. The reaction was stopped with stop solution, and absorbance was measured at 450 nm using a microplate reader. Insulin concentrations were calculated based on a standard curve generated from known standards. All as-says were conducted in duplicate to ensure accuracy and reproducibility.

Homeostasis model assessment of insulin resistance (HOMA-IR) was calculated using the following formula:

HOMA-IR = (FBG (mmol/L) × FINS (mIU/L))/22.5.

where FBG represents fasting blood glucose and FINS denotes fasting serum insulin levels. This index was used to evaluate systemic insulin resistance in each experimental group.

### 2.4 RNA extraction and quantitative RT-PCR analysis

Total RNA was extracted from approximately 50 mg of mouse liver tissue using the TransZol Up Plus RNA extraction kit (TransGen Biotech, Beijing, China), strictly adhering to the manufacturer’s guidelines. Tissue homogenization was performed thoroughly in pre-cooled reagent, and the resultant homogenates underwent standard chloroform phase separation, isopropanol precipitation, and ethanol washes. Purified RNA pellets were dissolved in RNase-free water and subsequently assessed for con-centration using a NanoDrop 2000 spectrophotometer (Thermo Fisher Scientific, Wilmington, DE, USA).

For quantitative real-time PCR (RT-qPCR), RNA samples with optical density ratios (260/280 nm and 260/230 nm) indicative of high purity (≥1.9) were selected. Re-verse transcription reactions were performed using the Quantscript RT-PCR kit (Tiangen Biotech, Beijing, China), employing random primers and oligo (dT) primers according to standard protocols provided by the manufacturer. The resulting cDNA samples were diluted appropriately for subsequent amplification.

Gene expression analysis was carried out using specific primer pairs synthesized by Sangon Biotech Co., Ltd. (Shanghai, China), detailed sequences of which are summarized in **Supplementary Table A1**. Real-time PCR amplifications were conducted in triplicate on an Applied Biosystems quantitative PCR system (Bio-Gener Technology Co., Ltd, Hangzhou, China). Reaction mixtures comprised SYBR Green fluorescent dye, template cDNA, forward and reverse primers, and RNase-free water to a final volume as per the manufacturer’s instructions. PCR cycling parameters were as follows: initial denaturation at 95 °C for 5 min, followed by 40 cycles of 95 °C for 15 s and 60 °C for 30 s, with melting curves generated to confirm the specificity of amplified products.

Relative gene expression levels were quantified using the comparative Ct method (2−ΔΔCt), and data were normalized against β-actin expression, selected as the stable in-ternal control gene.

### 2.5 Western Blot Analysis

In this experiment, total cellular proteins were extracted using a RIPA lysis buffer (supplemented with protease inhibitors) and quantified by the BCA method to ensure equal loading (approximately 20-30 µg per lane). After denaturation in a 5× SDS loading buffer, the samples were separated by SDS-PAGE on a 12% gel, followed by transfer onto a PVDF membrane pre-activated in 95% methanol. The membrane was blocked with 5% non-fat milk in TBST for 1 hour, then incubated overnight at 4 °C with primary antibodies specific to PPARα, IL-6, NF-κB, and LDL-R. Subsequently, HRP-conjugated secondary antibodies were applied for 1 hour at room temperature, and protein bands were visualized using an ECL chemiluminescent substrate, followed by image acquisition with a gel imaging system for quantitative analysis.

### 2.6 Histological analysis and lipid visualization

Liver, pancreas, and colon tissues were harvested from experimental mice and immediately fixed in 4% paraformaldehyde solution (Servicebio, Wuhan, China). For paraffin embedding, tissues underwent routine dehydration in graded ethanol, clearing with environmentally-friendly dewaxing solution, embedding, and subsequent sectioning at 4 µm thickness using a Leica RM2016 microtome (Leica, China). Liver samples designated for lipid analysis were embedded in optimal cutting temperature (OCT) compound, snap-frozen, and sectioned at 8 µm thickness on a Cryostar NX50 cryostat (Thermo Fisher, USA).

For hematoxylin-eosin (HE) staining, paraffin sections were dewaxed, rehydrated, stained with hematoxylin for 5 min, differentiated, blued in ammonia solution, and counterstained briefly with eosin for 15 s. Sections were dehydrated, cleared, and permanently mounted with neutral resin. Images were captured using a Nikon Eclipse E100 microscope equipped with a DS-U3 imaging system (Nikon, Japan). Nuclei were visualized as blue and cytoplasm as varying shades of pink.

For Oil Red O staining, liver cryosections were fixed with 4% paraformaldehyde for 15 min, followed by staining in freshly prepared Oil Red O working solution for 10 min at 4 °C. Differentiation was briefly conducted in 60% isopropanol, and nuclei were counterstained with hematoxylin. Slides were mounted with glycerol gelatin (Service-bio). Lipid droplets were visualized as bright orange-red against blue nuclei. Pancreatic and colonic tissues underwent identical HE staining procedures. All histopathological evaluations were performed independently by blinded observers.

### 2.7 Full-length 16S rRNA sequencing

Fecal microbial DNA was isolated using established protocols as previously outlined (48). The entire 16S rRNA gene spanning regions V1–V9 was amplified by PCR with primers 27F (5’-AGRGTTYGATYMTGGCTCAG-3’) and 1492R (5’-RGYTACCTTGTTACGACTT-3’). Following amplification, PCR products were evaluated on 2% agarose gels, purified according to the Pacific Biosciences guidelines, and subsequently utilized to generate SMRTbell libraries through blunt-end ligation. Amplicon sequencing was performed commercially by Shanghai Biozeron Biotechnology Co., Ltd. (Shanghai, China).

### 2.8 Bioinformatics analysis

Perform quality control using FastQC. The raw sequences were processed using QIIME2 (version 2023.05, https://docs.qiime2.org/). The further analysis, including noise reduction, correcting marginal sequence errors, removing chimeric sequences, removing singletons, joining paired-end reads, and dereplication, was achieved using DADA2 plugin. ASV (amplicon sequence variants) were defined based on 100% sequence similarity between the sequences. The species annotation results were based on the SILVA (version 138.1) (http://www.arb-silva.de/). The normalized ASV abundance data was subjected to alpha-diversity analysis, beta-diversity analysis, difference analysis and ecological network analysis using R (version 4.1.2). The Vegan package (version 2.6–6.1) was used to calculate the alpha-diversity and beta diversity. The ggplot2 package (version 3.5.0) was used to visualization.

### 2.9 Statistical analysis

Statistical analyses were conducted using GraphPad Prism software (version 9.0; GraphPad Software Inc., San Diego, CA, USA). Data are expressed as mean ± standard error of the mean (SEM). Group comparisons were performed using one-way analysis of variance (ANOVA), followed by Tukey’s multiple comparison test to assess inter-group differences. A two-tailed p-value < 0.05 was considered statistically significant. All statistical procedures adhered to established guidelines to ensure analytical robustness and reproducibility.

## 3 Results

### 3.1 Identification and mass spectral characterization of compounds in *Coriandrum sativum* seed aqueous extract

**Figure 1:**
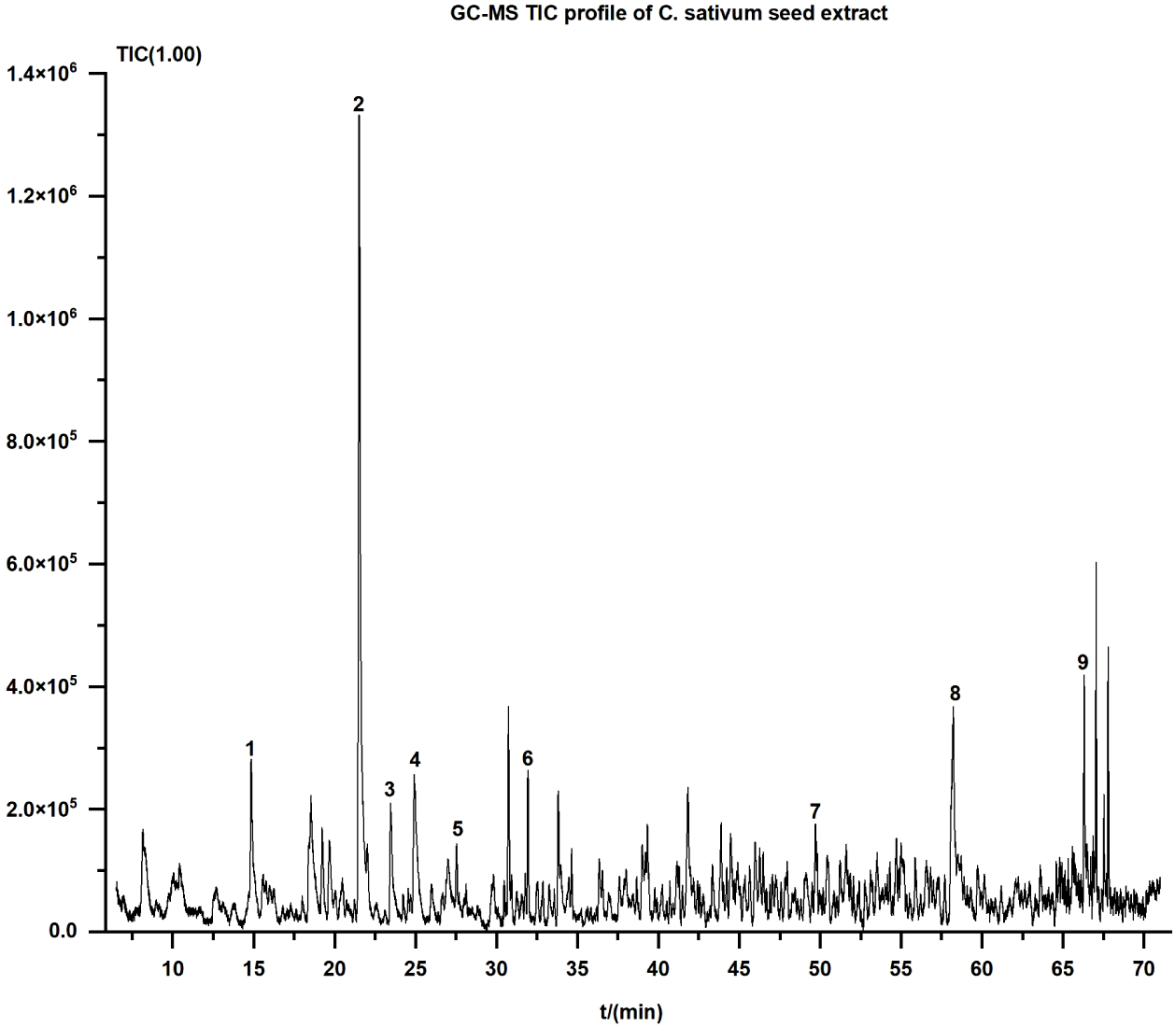
Total ion chromatogram (TIC) of the aqueous extract of *Coriandrum sativum* seeds obtained by GC–MS. Representative total ion chromatogram (TIC) of the aqueous extract of *Coriandrum sativum* seed acquired by gas chromatography–mass spectrometry (GC-MS). Peaks labeled 1 to 9 correspond to the major volatile constituents identified based on retention time and mass spectral matching with standard libraries. The x-axis represents retention time (min), and the y-axis indicates total ion current intensity (arbitrary units). Identified compounds include (1): 2,6-dimethylocta-3,7-diene-2,6-diol (2), 4-ethenyl-2-methoxyphenol (3), 3-methylhepta-1,6-dien-3-ol (4), (2E)-2,6-dimethylocta-2,7-diene-1,6-diol (5), dodecanal (6), hexadecane (7), 2,6,10,14-tetramethylhexadecane (8), hexadecanoic acid, and (9) octadecanoic acid. Abbreviation: GC-MS, gas chromatography–mass spectrometry.

The chemical composition of the aqueous extract of *Coriandrum sativum* seeds was characterized by gas chromatography–mass spectrometry (GC–MS). A total of nine compounds were identified based on retention time, retention index (RI), molecular formula, and mass spectral fragmentation patterns (**Figure 1**; **Table 1**). Compound identities were further supported by similarity index (SI) values and confirmed through comparison with standard mass spectral libraries (**Table 2**). Representative electron ionization (EI) mass spectra of these nine compounds, including their characteristic fragment ions and chemical structures, are presented in (**Supplementary Figure A1**). Each spectrum displays the mass-to-charge ratio (m/z) and relative intensity of key fragment ions, providing additional structural confirmation for compound identification.

**Table 1:** GC-MS analytical parameters of identified compounds in *C. sativum* seed extract.

Number	Compounds	Retention time (min)	Retention index	Formula
1	2,6-dimethylocta-3,7-diene-2,6-diol	14.837	1197	C_10_H_18_O_2_
2	4-ethenyl-2-methoxyphenol	21.522	1293	C_9_H_10_O_2_
3	3-methylhepta-1,6-dien-3-ol	23.453	888	C_8_H_14_O
4	(2E)-2,6-dimethylocta-2,7-diene-1,6-diol	24.923	1325	C_10_H_18_O_2_
5	Dodecanal	27.528	1402	C_12_H_24_O
6	Hexadecane	31.952	1612	C_16_H_34_
7	2,6,10,14-tetramethylhexadecane	49.709	1753	C_20_H_42_
8	Hexadecanoic acid	58.223	1968	C_16_H_32_O_2_
9	Octadecanoic acid	66.304	2167	C_18_H_36_O_2_

The retention time, retention index, and molecular formula of compounds in C. sativum seed aqueous extract were determined by gas chromatography–mass spectrometry (GC-MS). All compounds were identified based on comparison with standard mass spectral libraries. No internal standards were applied. GC-MS, gas chromatography–mass spectrometry.

**Table 2:** Spectral matching and relative abundance of characterized compounds in *C. sativum* seed extract.

Number	Compounds	CAS	SI	Conc./%
1	2,6-dimethylocta-3,7-diene-2,6-diol	13741-21-4	93	1.35
2	4-ethenyl-2-methoxyphenol	7786-61-0	94	11.62
3	3-methylhepta-1,6-dien-3-ol	34780-69-3	90	1.08
4	(2E)-2,6-dimethylocta-2,7-diene-1,6-diol	64142-78-5	94	2.58
5	Dodecanal	112-54-9	95	0.51
6	Hexadecane	544-76-3	94	1.1
7	2,6,10,14-tetramethylhexadecane	638-36-8	90	1.47
8	Hexadecanoic acid	57-10-3	94	6.13
9	Octadecanoic acid	57-11-4	91	4.28

Among the identified constituents, monoterpenoid alcohols included 2,6-dimethylocta-3,7-diene-2,6-diol (Compound 1, RI 1197) and its structural isomer (2E)-2,6-dimethylocta-2,7-diene-1,6-diol (Compound 4, RI 1325), with relative abundances of 1.35% and 2.58%, respectively. These compounds shared major fragment ions at m/z 43 and 67, attributed to cleavage of the terminal hydroxylated isoprenoid chains. Their spectral profiles were distinguishable by the presence of additional peaks such as m/z 93 and 152 in Compound 4.

Phenolic and unsaturated alcohol derivatives were represented by 4-ethenyl-2-methoxyphenol (Compound 2, RI 1293) and 3-methylhepta-1,6-dien-3-ol (Compound 3, RI 888). Compound 2 was the most abundant component (11.62%), showing diagnostic ions at m/z 150, 107, and 77, consistent with fragmentation of a vinyl-substituted guaiacol core. Compound 3 presented a simpler fragmentation pat-tern dominated by m/z 71 and 43.

Compounds 5 to 7 included dodecanal (RI 1402), hexadecane (RI 1612), and 2,6,10,14-tetramethylhexadecane (RI 1753), with relative abundances ranging from 0.51% to 1.47%. Dodecanal produced characteristic aldehyde fragments at m/z 57 and 82, while the two saturated hydrocarbons showed typical alkyl ion series, including m/z 57, 71, and 99.

Long-chain fatty acids were identified as hexadecanoic acid (Compound 8, RI 1968; 6.13%) and octadecanoic acid (Compound 9, RI 2167; 4.28%). Both compounds exhibited typical McLafferty rearrangement ions at m/z 60 and 73, in addition to sequential β-cleavage fragments such as m/z 129 and 143. Their spectral data were in agreement with reference spectra of saturated fatty acids.

The complete fragmentation ion distribution and intensity values for all compounds are provided in **Supplementary Table A2**. The identification of structurally diverse compounds, including monoterpenoids, phenolics, aldehydes, alkanes, and fatty acids, establishes the chemical basis for further pharmacological evaluation of the extract.

### 3.2 *Coriandrum sativum* seed extract attenuates diet-induced weight gain and visceral adiposity

**Figure 2:**
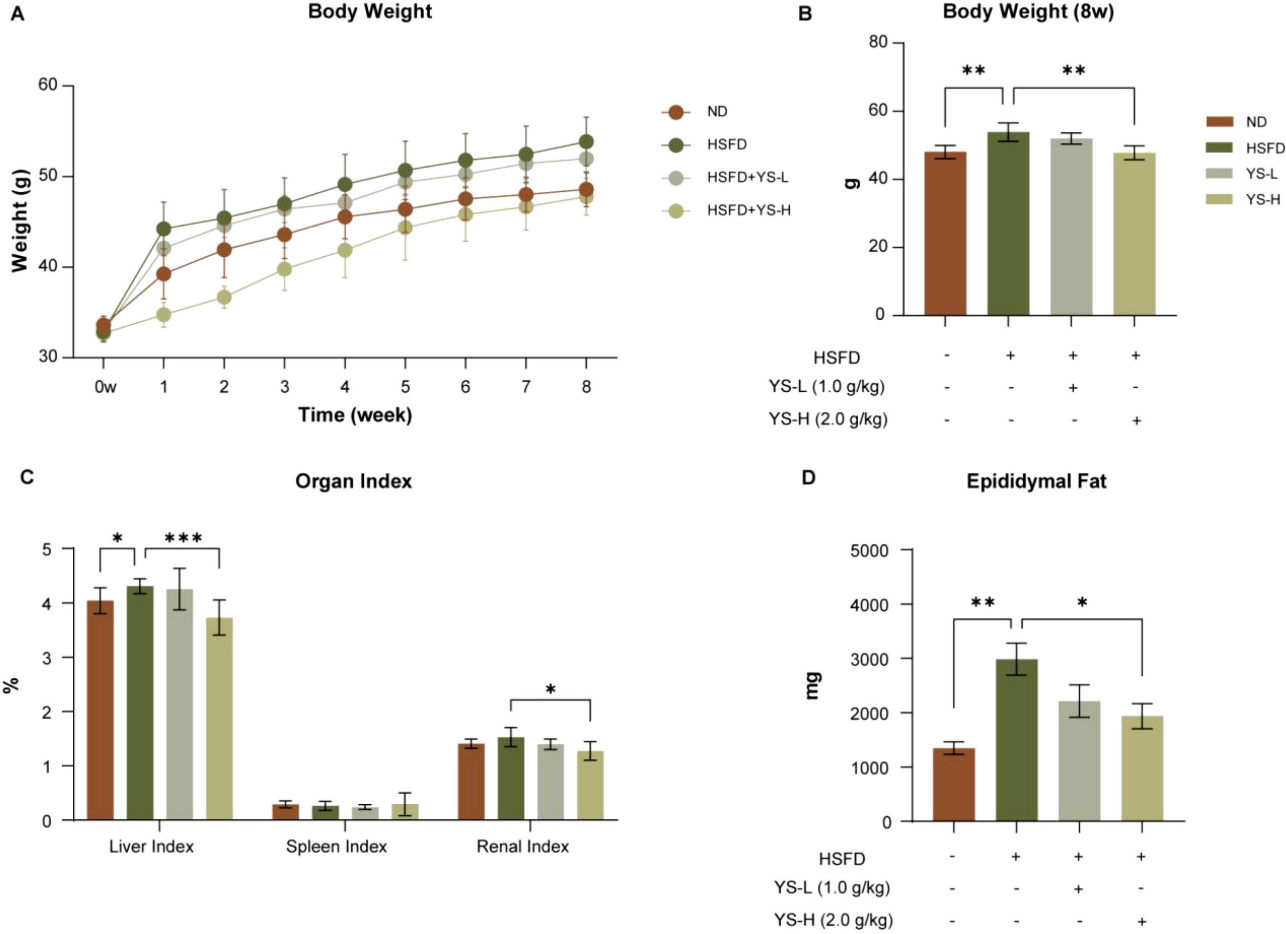
Impact of *Coriandrum sativum* seed aqueous extract on body weight trajectory, organ indices, and visceral adiposity in mice following an 8-week HSFD intervention. **(A)** Weekly body weight changes over an 8-week period. **(B)** Final body weight at week 8. **(C)** Organ indices, including liver, spleen, and kidney weights expressed as a percentage of total body weight. **(D)** Epididymal fat mass. Mice fed a high-sugar, high-fat diet (HSFD) showed significant increases in body weight, liver index, and epididymal fat compared with the normal diet (ND) group. Oral administration of *C. sativum* seed aqueous extract at low (YS-L, 1.0 g/kg) and high (YS-H, 2.0 g/kg) doses dose-dependently attenuated these parameters. Data are expressed as mean ± SEM (*n* = 7). Statistical significance: *p < 0.05, **p < 0.01, ***p < 0.001. HSFD, high-sugar high-fat diet; ND, normal diet; YS-L, low dose *C. sativum* extract; YS-H, high dose extract; SEM, standard error of the mean.

Following phytochemical identification, we evaluated the physiological impact of *Coriandrum sativum* seed extract in a murine model of metabolic dysfunction. As illustrated in **Figure 2A**, mice receiving HSFD exhibited a continuous elevation in body weight across the 8-week period, markedly exceeding that of the ND group. In contrast, mice treated with YS, particularly at the higher dose, showed a gradual attenuation of weight gain beginning at week 3, with a clearer divergence observed from week 5 onward.

Final body weights recorded at week 8 (**Figure 2B**) reinforced the longitudinal findings. HSFD-fed mice displayed significantly higher body weights compared to ND animals (*p* < 0.01), while both YS-L and YS-H groups demonstrated weight reductions. The effect was more pronounced in the YS-H group (*p* < 0.01 vs. HSFD), suggesting a potential dose-dependent response.

Analysis of organ indices (**Figure 2C**) revealed that HSFD led to liver enlargement, evidenced by a significant increase in liver index compared to ND (*p* < 0.05). This elevation was substantially diminished by YS administration, with YS-H reducing liver index values to near-baseline levels (*p* < 0.001 vs. HSFD). While spleen indices remained largely unaltered, the renal index was moderately elevated in the HSFD group and significantly reduced in YS-H-treated mice (*p* < 0.05), indicating a potential normalization of renal mass.

As shown in **Figure 2D**, HSFD significantly elevated epididymal fat mass to ND controls (*p* < 0.01), consistent with visceral adipose accumulation. Both YS-treated groups exhibited reductions in fat mass, with YS-H achieving a statistically significant decrease compared to HSFD alone (*p* < 0.05). These reductions paralleled the observed suppression of body weight gain.

Together, these findings establish a physiological basis for the metabolic effects of the extract, highlighting its capacity to mitigate HSFD-induced weight gain, hepatomegaly, renal index elevation, and visceral fat expansion in a dose-responsive manner.

### 3.3 *Coriandrum sativum* seed extract improves glucose metabolism and preserves pancreatic structure

**Figure 3:**
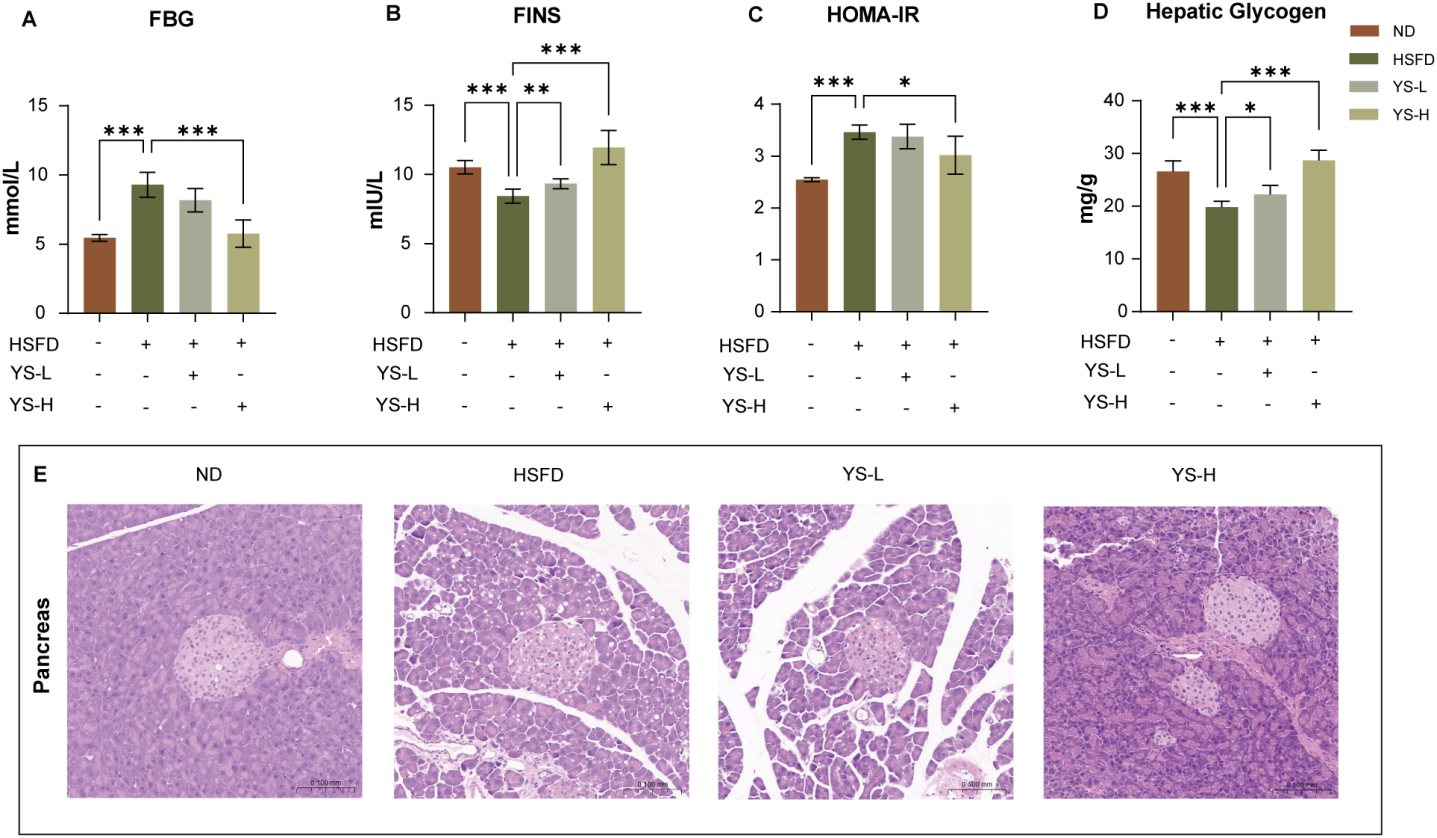
*Coriandrum sativum* seed aqueous extract attenuates hyperglycemia and improves pancreatic histomorphology in HSFD-fed mice. **(A)** Fasting blood glucose (FBG); **(B)** Fasting insulin (FINS); **(C)** Homeostatic model assessment of insulin resistance (HOMA-IR); **(D)** Hepatic glycogen content; **(E)** Representative HE-stained pancreatic sections from each group. Compared with normal diet (ND) mice, HSFD-fed mice exhibited significant hyperglycemia, hyperinsulinemia, increased HOMA-IR index, and reduced hepatic glycogen content. Histological assessment revealed disrupted islet architecture and acinar atrophy in the HSFD group. Administration of *C. sativum* extract at both low (YS-L, 1.0 g/kg) and high doses (YS-H, 2.0 g/kg) markedly improved these metabolic and histological alterations. Data are presented as mean ± SEM (*n* = 7). Statistical significance: *p < 0.05, **p < 0.01, ***p < 0.001. FBG, fasting blood glucose; FINS, fasting insulin; HOMA-IR, homeo-static model assessment of insulin resistance; HSFD, high-sugar high-fat diet; ND, normal diet; YS-L, low-dose C. sativum extract; YS-H, high-dose extract; HE, hematoxylin and eosin.

Given the extract’s effectiveness in reducing HSFD-induced weight gain and adiposity, we next evaluated its role in glycemic regulation and pancreatic integrity—two closely linked endpoints of metabolic dysfunction (**Figures 3A–E**).

Consistent with the obesity-related metabolic burden observed in the previous section, fasting blood glucose levels were markedly elevated in HSFD-fed mice relative to the normal diet group (*p* < 0.001, **Figure 3A**). Both low and high doses of *Coriandrum sativum* seed extract reduced glucose levels, with the high-dose group restoring normoglycemia to a greater extent (*p* < 0.001 vs. HSFD). Parallel changes were observed in circulating insulin concentrations (**Figure 3B**). HSFD mice exhibited marked hyperinsulinemia (*p* < 0.001), while extract treatment attenuated this elevation in a dose-responsive manner (*p* < 0.01 and *p* < 0.001 for low and high doses, respectively), suggesting improved insulin resistance.

The homeostasis model assessment of insulin resistance (HOMA-IR) further sup-ported these findings (**Figure 3C**). HSFD feeding led to a significant increase in HOMA-IR values (*p* < 0.001), reflecting systemic insulin resistance. Treatment with *Coriandrum sativum* seed extract reduced HOMA-IR scores, with the high-dose group demonstrating a significant improvement (*p* < 0.05 vs. HSFD), indicating partial restoration of insulin responsiveness.

In line with these improvements, hepatic glycogen stores—often depleted under insulin-resistant conditions—were significantly reduced in HSFD-fed mice (*p* < 0.001 vs. ND, **Figure 3D**). Both extract-treated groups exhibited a restoration of glycogen content, with the high-dose group achieving greater recovery (*p* < 0.05 vs. HSFD; *p* < 0.001 vs. HSFD), suggesting enhanced hepatic glucose utilization and storage.

Histological assessment of pancreatic tissue provided morphological evidence supporting the observed metabolic improvements (**Figure 3E**). In ND-fed mice, hematoxylin and eosin staining revealed well-defined islet architecture with intact cellular boundaries and uniform acinar distribution. In contrast, HSFD-fed mice exhibited marked islet distortion, including irregular borders, vacuolar degeneration, and disorganized exocrine lobules, indicative of diet-induced pancreatic injury. Notably, both extract-treated groups demonstrated attenuation of these pathological features. In the high-dose group, the islets retained their structural integrity with reduced cellular swelling and more regular contours, while the surrounding acinar cells showed improved alignment and nuclear morphology, approaching the histological presentation of the ND group. Collectively, these data reveal that *Coriandrum sativum* seed extract not only reduces hyperglycemia and insulin resistance but also mitigates the downstream hepatic and pancreatic consequences of prolonged HSFD exposure. This functional transition from systemic indicators to organ-level protection supports its broader therapeutic potential in metabolic regulation.

### 3.4 *Coriandrum sativum* seed extract alleviates hepatic lipid accumulation and liver injury

**Figure 4:**
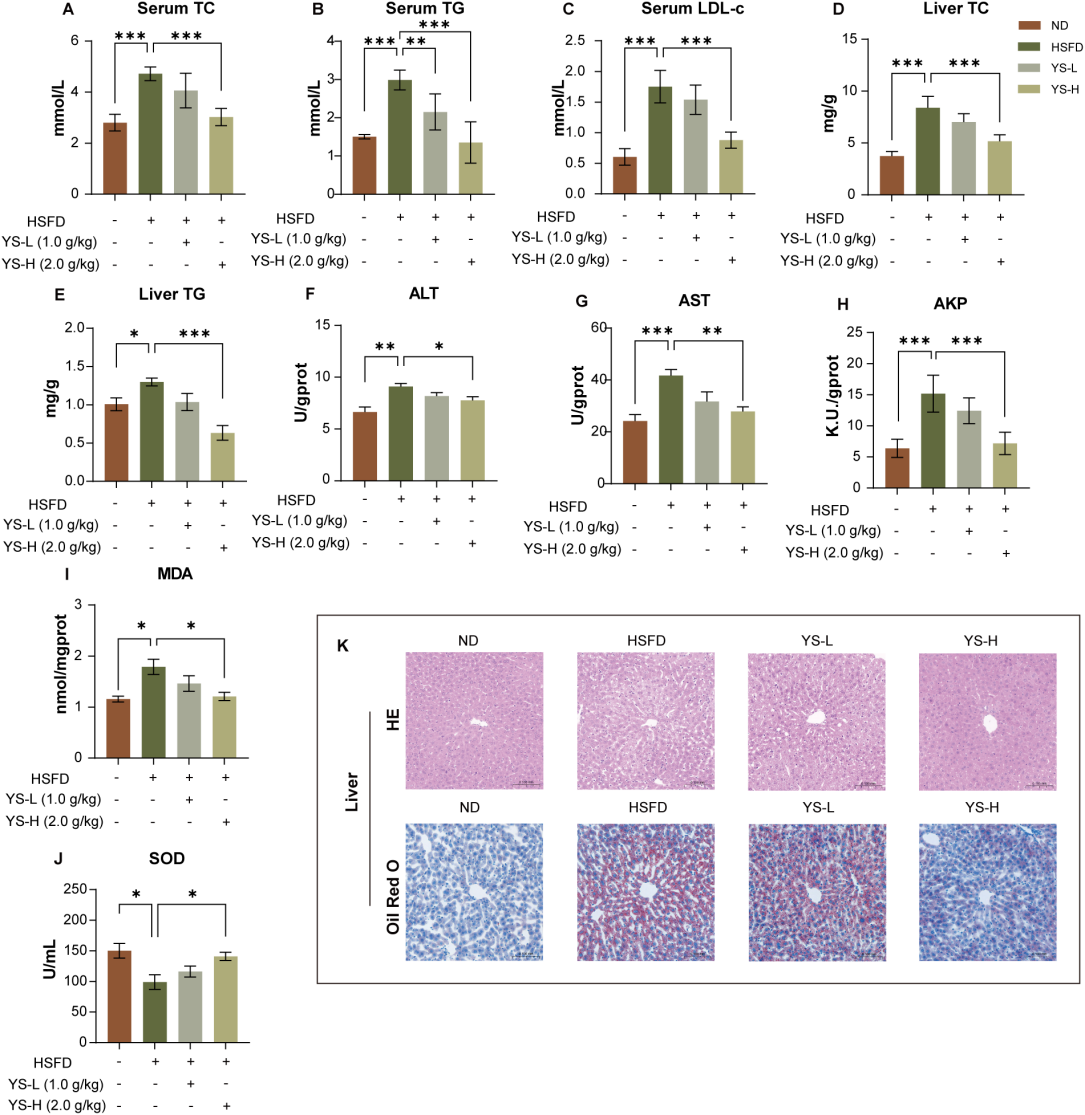
*Coriandrum sativum* seed extract ameliorates dyslipidemia, hepatic steatosis, liver injury, and oxidative stress in HSFD-fed mice. **(A–C)** Serum total cholesterol (TC), triglycerides (TG), and low-density lipoprotein cholesterol (LDL-c); **(D, E)** Hepatic TC and TG content; **(F–H)** Hepatic alanine aminotransferase (ALT), aspartate aminotransferase (AST), and alkaline phosphatase (AKP) activities; **(I, J)** Hepatic malondialdehyde (MDA) level and superoxide dismutase (SOD) activity. **(K)** Representative hematoxylin and eosin (HE) and Oil Red O-stained liver sections. Mice fed a high-sugar high-fat diet (HSFD) exhibited marked dyslipidemia, hepatic lipid accumulation, liver injury, and oxidative stress, as evidenced by elevated serum lipids, ALT, AST, MDA levels, and decreased hepatic SOD activity. Histological sections revealed hepatocyte ballooning and intracellular lipid droplet accumulation. Treatment with C. sativum extract (YS-L: 1.0 g/kg; YS-H: 2.0 g/kg) dose-dependently improved serum and hepatic lipid profiles, reduced liver enzyme levels, restored redox balance, and ameliorated hepatic histopathological alterations. Data are expressed as mean ± SEM (*n* = 7). Statistical significance: *p < 0.05, **p < 0.01, ***p < 0.001. TC, total cholesterol; TG, triglycerides; LDL-c, low-density lipoprotein cholesterol; ALT, alanine aminotransferase; AST, aspartate aminotransferase; AKP, alkaline phosphatase; MDA, malondialdehyde; SOD, super-oxide dismutase; HE, hematoxylin and eosin.

Following improvements in glucose homeostasis and pancreatic morphology, we next examined the effects of *Coriandrum sativum* seed extract on hepatic lipid metabolism, liver enzyme activities, oxidative stress markers, and histopathological features in HSFD-fed mice (**Figures 4A–K**).

Mice subjected to HSFD exhibited significant dyslipidemia, with elevated serum total cholesterol (TC), triglycerides (TG), and low-density lipoprotein cholesterol (LDL-C) levels compared to the normal diet group (*p* < 0.001, **Figures 4A–C**). Both extract-treated groups showed dose-dependent reductions in these lipid markers, with the high-dose group achieving the most pronounced effects (TC: *p* < 0.001; TG: *p* < 0.001; LDL-C: *p* < 0.001 vs. HSFD), clearly indicating effective systemic lipid-lowering activity.

Consistent with serum profiles, hepatic lipid accumulation was also aggravated in HSFD-fed mice, as evidenced by increased liver TC and TG levels (*p* < 0.001 vs. ND, **Figures 4D, E**). Treatment with *Coriandrum sativum* seed extract significantly attenuated hepatic lipid content in a dose-dependent manner. The high-dose group demonstrated significantly greater reductions in hepatic TC and TG compared to the low-dose group (*p* < 0.01), approaching near-baseline values.

Liver enzyme activities were further assessed to evaluate hepatic injury. Serum levels of alanine aminotransferase (ALT), aspartate aminotransferase (AST), and alkaline phosphatase (AKP) were all significantly elevated in HSFD-fed mice (**Figures 4F–H**), indicating hepatocellular damage. Both low- and high-dose extract treatments ameliorated these elevations, with the high-dose group showing significantly greater reductions in ALT (*p* < 0.05), AST (*p* < 0.01), and AKP (*p* < 0.001 vs. HSFD), demonstrating a clear dose-dependent protection against diet-induced hepatic injury.

To further investigate hepatic oxidative status, malondialdehyde (MDA) and superoxide dismutase (SOD) were measured (**Figures 4I, J**). HSFD feeding significantly increased hepatic MDA levels (*p* < 0.05) while decreasing SOD activity (*p* < 0.05), indicating enhanced lipid peroxidation and weakened antioxidant capacity. Extract administration partially reversed these effects, with the high-dose group demonstrating significantly greater reductions in MDA and improvements in SOD levels compared to the low-dose group (*p* < 0.05), supporting a dose-dependent antioxidative role of the extract.

Histological analysis of liver tissues further substantiated the biochemical findings. In HE-stained sections (**Figure 4K**, top row), livers from ND-fed mice displayed normal hepatic architecture with clear hepatic cords, intact nuclei, and regular sinusoidal structure. HSFD-fed mice, however, exhibited notable pathological alterations, including hepatocellular ballooning, cytoplasmic rarefaction, and sinusoidal congestion, indicative of steatosis and early inflammatory response. Treatment with *Coriandrum sativum* seed extract ameliorated these histopathological changes in a clear dose-dependent manner. Livers from the high-dose group demonstrated significantly greater restoration of hepatic cord alignment, reduction in cytoplasmic vacuolation, and normalization of sinusoidal spacing compared to the low-dose group.

Oil Red O staining (**Figure 4K**, bottom row) revealed minimal lipid deposition in ND livers, whereas HSFD induced prominent macrovesicular steatosis, characterized by widespread lipid droplet accumulation within hepatocyte cytoplasm. Extract treatment markedly reduced lipid deposition, with the high-dose group showing significantly greater decreases in intracellular lipid accumulation and improved lobular architecture compared to the low-dose group, clearly demonstrating a dose-dependent capacity to mitigate hepatic steatosis. These results demonstrate that *Coriandrum sativum* seed extract effectively mitigates hepatic lipid accumulation, reduces biochemical markers of liver injury, enhances antioxidant defense, and preserves hepatic histomorphology under HSFD challenge, further supporting its multi-targeted hepatoprotective potential.

### 3.5 *Coriandrum sativum* seed extract modulates hepatic expression of lipid metabolism and inflammation-related genes

Given the observed improvements in hepatic lipid accumulation, liver enzyme activities, and histopathological features, hepatic gene expression profiling was conducted to further characterize the molecular responses induced by *Coriandrum sativum* seed extract. Quantitative RT-PCR was performed on liver tissues from the high-dose group (2.0 g/kg), which consistently showed superior biochemical and morphological improvements in previous analyses.

**Figure 5:**
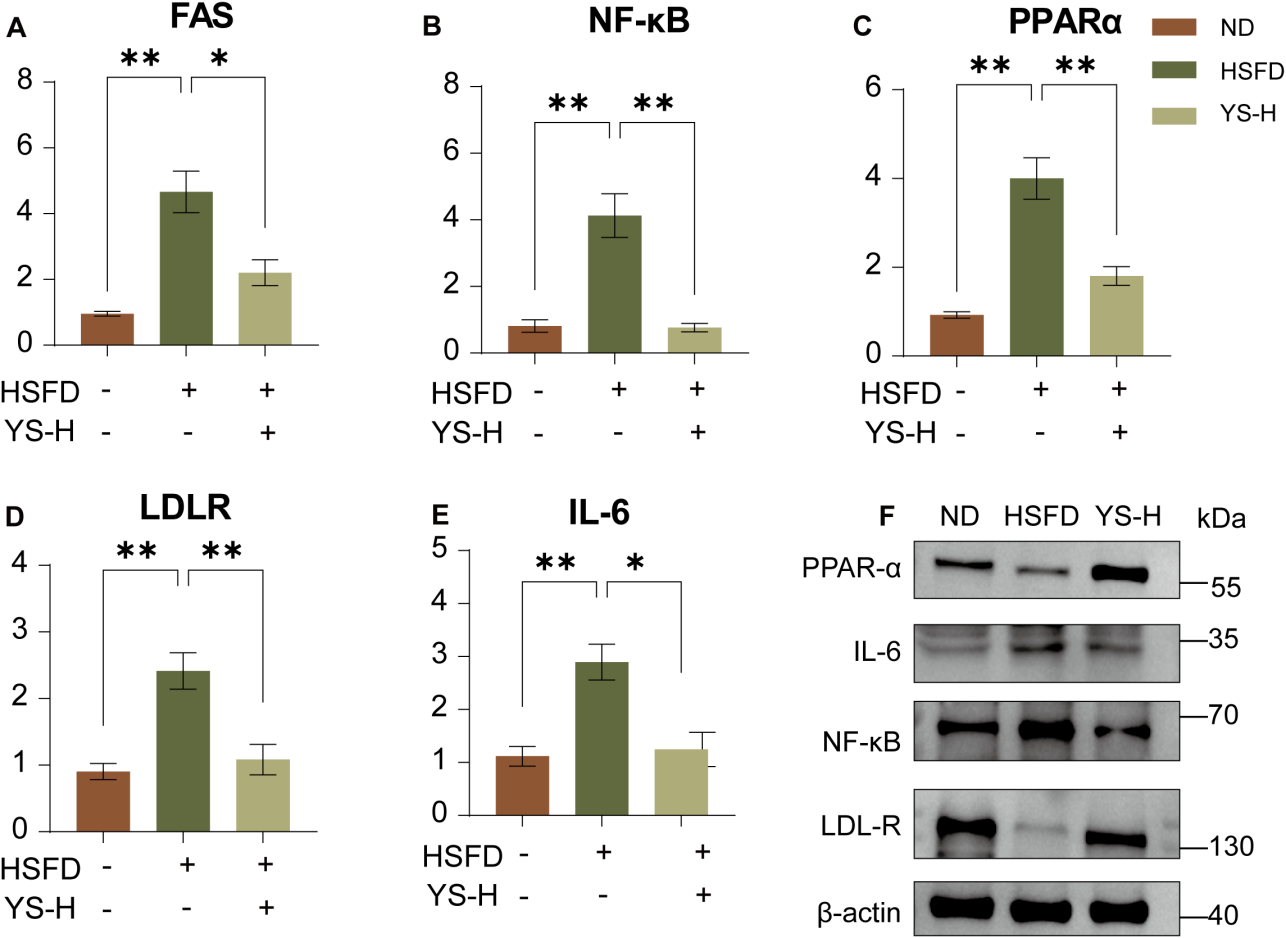
Effects of *Coriandrum sativum* seed extract on hepatic expression of lipid metabolism and inflammatory genes in HSFD-fed mice. Relative mRNA expression levels of **(A)** fatty acid synthase (FAS), **(B)** nuclear factor-kappa B (NF-κB), **(C)** peroxisome proliferator-activated receptor alpha (PPARα), **(D)** low-density lipoprotein receptor (LDLR), and **(E)** interleukin-6 (IL-6) in liver tis-sues, as measured by quantitative real-time PCR (RT-PCR). **(F)** Representative Western blot bands for PPARα, IL-6, NF-κB, and LDLR proteins. Data are presented as mean ± SEM (*n* = 6). Statistical significance: *p < 0.05, **p < 0.01. FAS, fatty acid synthase; NF-κB, nuclear factor-kappa B; PPARα, peroxisome proliferator-activated receptor alpha; LDLR, low-density lipoprotein receptor; IL-6, interleukin-6; HSFD, high-sugar high-fat diet; ND, normal diet; YS-H, high-dose *C. sativum* extract; SEM, standard error of the mean.

High-sugar, high-fat feeding markedly disrupted the expression of lipid metabolic regulators. Hepatic mRNA levels of FAS, a central enzyme in *de novo* lipogenesis, were significantly upregulated in HSFD-fed mice compared to the ND group (*p* < 0.01; **Figure 5A**). Similarly, transcript levels of PPARα, which governs lipid oxidation, and LDLR, involved in cholesterol clearance, were both elevated under HSFD conditions (*p* < 0.01; **Figures 5C, D**), reflecting a shift in hepatic lipid handling toward a pathophysiological state. At the same time, markers of hepatic inflammation were markedly induced. The expression of NF-κB, a transcription factor activated in response to metabolic stress, was significantly increased (*p* < 0.01; **Figure 5B**), alongside elevated expression of IL-6, a downstream pro-inflammatory cytokine (*p* < 0.01; **Figure 5E**).

Intriguingly, Western blot analysis revealed a distinct regulatory pattern at the protein level: the protein expressions of both PPARα and LDLR were upregulated in the extract-treated group, a trend opposite to their mRNA changes. This divergence suggests that the extract may enhance the translational efficiency or stabilize the proteins of PPARα and LDLR, representing a sophisticated, multi-layered regulatory mechanism. For inflammatory markers, a consistent downregulatory trend was observed: both NF-κB and IL-6 were significantly downregulated at mRNA and protein levels after extract treatment, aligning with the reduced hepatic inflammatory burden (**Figure 5F**).

The coordinated expression profile observed in the high-dose group corresponds closely with the extract-induced improvements in hepatic lipid levels, oxidative stress, and tissue morphology. These multi-level changes reflect a complex and harmonized modulation of hepatocellular metabolic and immune pathways under dietary challenge, where the upregulation of key functional proteins may be crucial for restoring metabolic homeostasis.

### 3.6 *Coriandrum sativum* seed extract preserves colonic histomorphology under HSFD challenge

Building upon the hepatic transcriptomic evidence of reduced inflammation and metabolic stress, we further evaluated the effects of *Coriandrum sativum* seed extract on intestinal tissue architecture, given the well-established gut–liver axis and its relevance in metabolic disease progression. Morphological assessment of hematoxylin and eosin-stained colonic sections was performed to determine whether dietary intervention could preserve mucosal structure under long-term HSFD exposure (**Supplementary Figure A2**).

Colon tissue from ND-fed mice exhibited normal histological features, including intact mucosal layers, tightly packed crypts, uniform epithelial cell arrangement, and well-distributed goblet cells. In contrast, HSFD-fed mice showed pronounced mucosal injury, characterized by crypt distortion, epithelial attenuation, increased submucosal spacing, and infiltration of inflammatory cells within the lamina propria—indicative of impaired intestinal barrier integrity.

Treatment with *Coriandrum sativum* seed extract attenuated these pathological changes in a dose-dependent manner. The low-dose group demonstrated partial restoration, with moderate improvements in crypt organization and epithelial density. Notably, the high-dose group displayed substantial morphological preservation, with nearly continuous epithelial lining, restored crypt depth, and normalization of goblet cell architecture, closely resembling the histological presentation observed in the ND group.

These structural findings indicate that *Coriandrum sativum* seed extract confers protective effects on colonic mucosa under metabolic challenge and provide morphological context for the subsequent investigation of gut microbiota composition.

### 3.7 *Coriandrum sativum* seed extract reshapes gut microbial diversity and community composition

**Figure 6:**
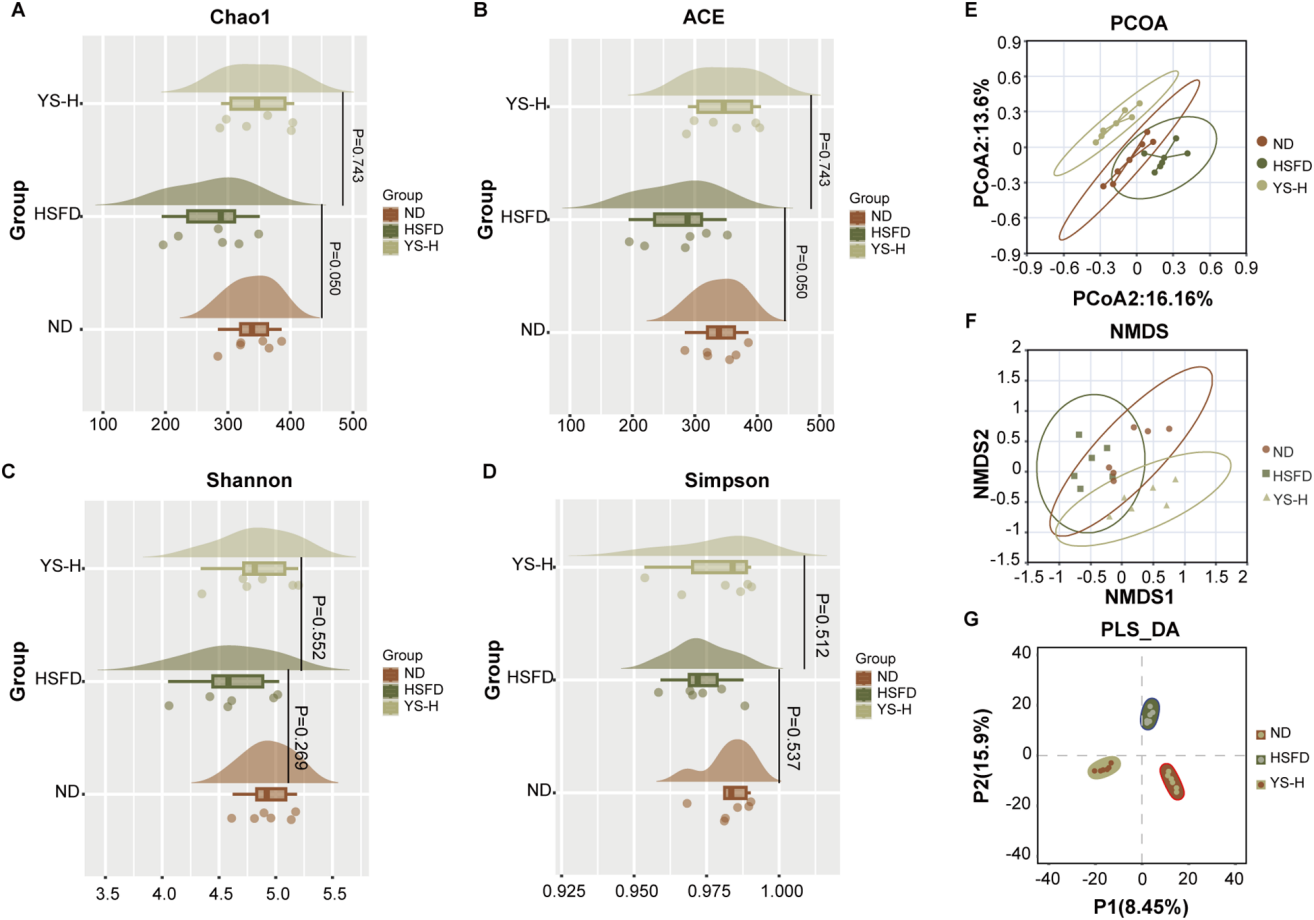
Alpha and beta diversity analyses of gut microbiota composition in mice fed normal diet (ND), high-sugar high-fat diet (HSFD), or HSFD supplemented with high-dose C. sativum seed extract (YS-H). **(A–D)** Alpha diversity indices, including Chao1, ACE, Shannon, and Simpson, were calculated to assess species richness and evenness among groups. **(E–G)** Beta diversity analysis based on principal coordinate analysis (PCoA), nonmetric multidimensional scaling (NMDS), and partial least squares discriminant analysis (PLS-DA) demonstrated distinct clustering of microbial communities. HSFD feeding resulted in reduced microbial diversity and altered community composition, while high-dose C. sativum extract (YS-H, 2.0 g/kg) partially restored microbial diversity and shifted overall community structure toward that of the ND group. PCoA, principal coordinate analysis; NMDS, nonmetric multidimensional scaling; PLS-DA, partial least squares discriminant analysis; HSFD, high-sugar high-fat diet; ND, normal diet; YS-H, high-dose *C. sativum* extract.

Considering the observable dose-related metabolic benefits, gut microbiota profiling was conducted in the high-dose *Coriandrum sativum* seed extract group (YS-H) to evaluate its potential role in reshaping intestinal microbial ecology under high-fat high-sugar diet (HSFD) conditions. Compared to the normal diet (ND) group, the HSFD group showed a downward trend in alpha diversity indices, which was partially restored by YS-H intervention, though none of the differences reached statistical significance. (**Figures 6A–D**). To better understand the broader community structure, β diversity was explored using principal coordinates analysis (PCoA), non-metric multidimensional scaling (NMDS), and partial least squares discriminant analysis (PLS-DA), each based on Bray–Curtis dissimilarity matrices (**Figures 6E–G**). A clear separation was observed between ND and HSFD groups, highlighting the extent of dysbiosis induced by dietary overload.

**Figure 7:**
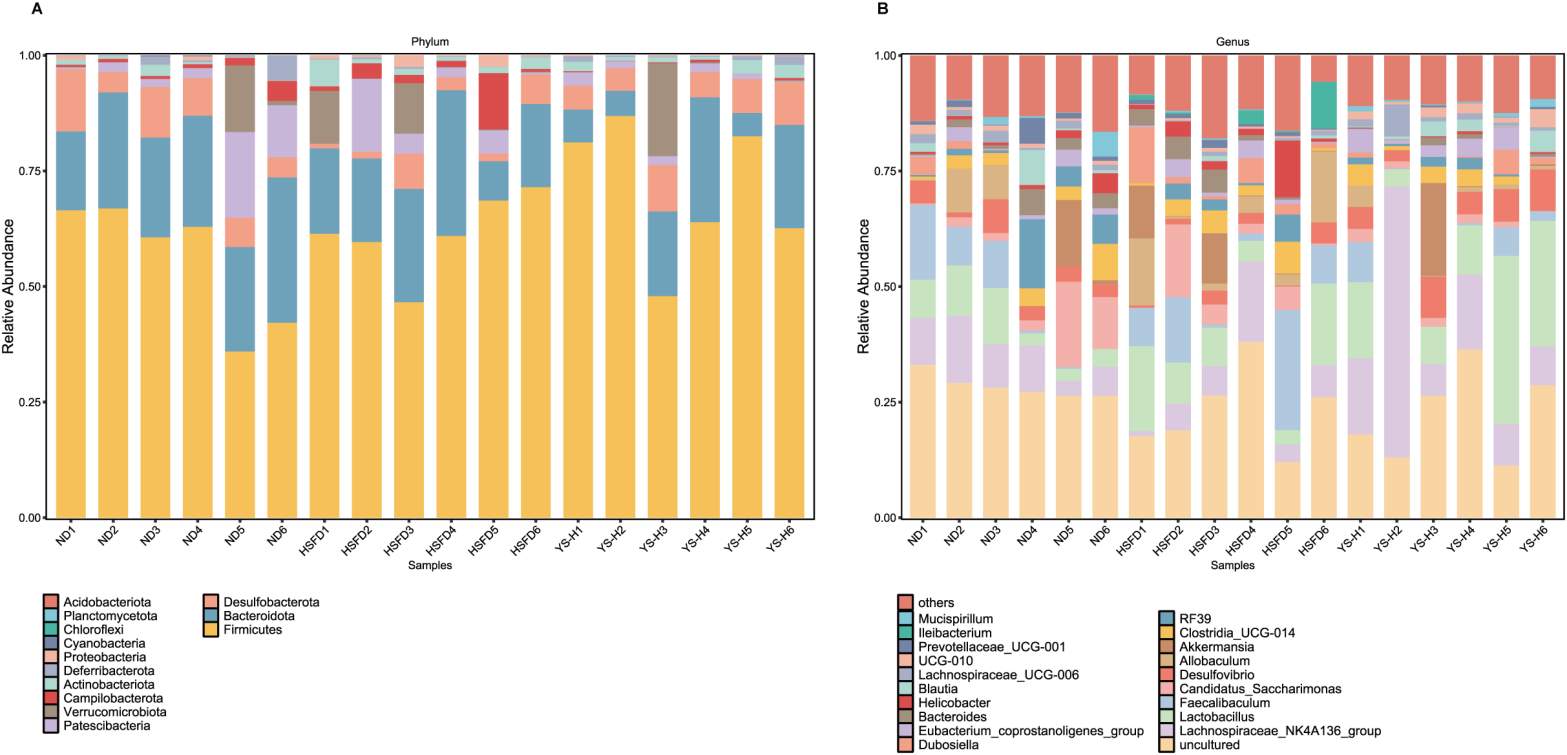
Taxonomic composition of gut microbiota in ND, HSFD, and YS-H groups at the phylum and genus levels. **(A, B)** Stacked bar plots showing the relative abundance profiles of the top 20 most abundant taxa at the phylum **(A)** and genus **(B)** levels across all samples. Distinct compositional shifts were observed in response to high-sugar high-fat diet (HSFD) feeding, while intervention with high-dose *C. sativum* seed extract (YS-H, 2.0 g/kg) altered the microbial community structure compared to the HSFD group. HSFD, high-sugar high-fat diet; ND, normal diet; YS-H, high-dose *C. sativum* extract.

Phylum-level microbial composition was assessed to determine dietary and intervention-induced shifts in dominant taxonomic groups (**Figure 7A**). Across all three groups, Firmicutes and Bacteroidota remained the predominant phyla. HSFD feeding resulted in a visible reduction in the relative abundance of Bacteroidota, accompanied by increased proportions of Desulfobacterota and Proteobacteria, as indicated by both column height and heatmap intensity. Verrucomicrobiota, which was undetectable in the HSFD group, was present in the ND group and reappeared in extract-treated mice. Actinobacteriota and Campylobacterota exhibited elevated levels in the HSFD group and lower abundance in YS-H mice. In the extract-treated group, the relative abundance of Desulfobacterota and Proteobacteria appeared lower than in the HSFD group, suggesting a directional shift in phylum-level structure following intervention, although Firmicutes remained dominant across all groups. At the genus level (**Figure 7B**), further analysis of the microbial community structure revealed significant shifts in the composition of dominant genera due to dietary intervention. Compared to the ND group, the HSFD group exhibited a marked reduction in the relative abundance of *Lactobacillus* and the *Lachnospiraceae*_NK4A136_group. Conversely, HSFD feeding resulted in a significant increase in the proportions of *Faecalibaculum* and *Desulfovibrio*, indicating specific diet-induced dysbiosis. Notably, these trends were partially reversed following intervention with *Coriandrum sativum* seed extract. Compared to the HSFD group, the abundance of Lactobacillus was restored, while the relative abundance of *Faecalibaculum* decreased. This suggests that the extract intervention helps regulate the genus-level composition of the gut microbiota, shifting it back toward a normal state.

### 3.8 *Coriandrum sativum* seed extract induces taxonomically distinct microbial signatures

**Figure 8:**
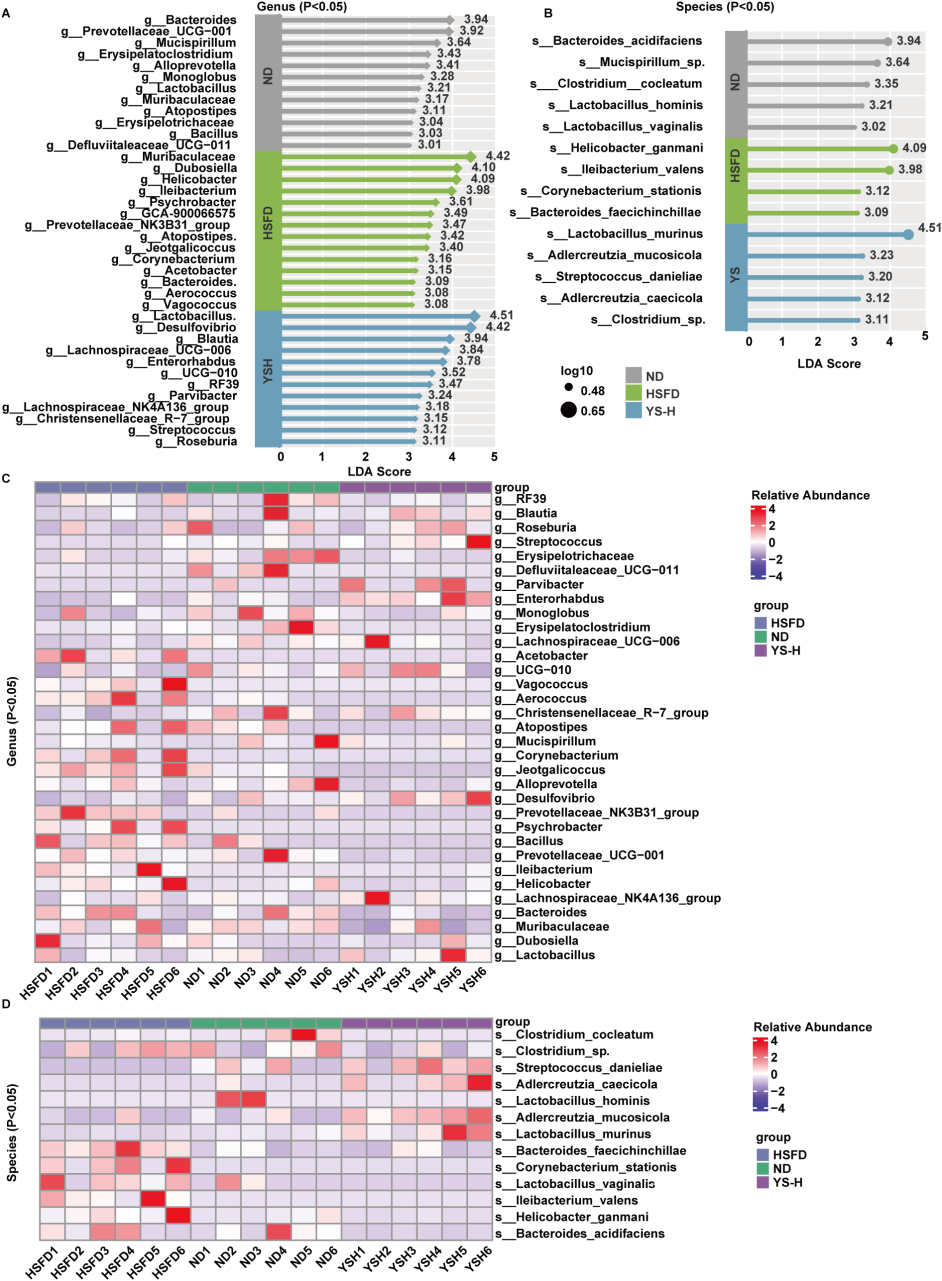
Differential bacterial taxa at the genus and species levels following *Coriandrum sativum* seed extract intervention in HSFD-fed mice. **(A, B)** Linear discriminant analysis (LDA) scores of significantly discriminative bacterial taxa at the genus **(A)** and species **(B)** levels, identified using LEfSe (LDA score > 3.0, *p* < 0.05). Taxa enriched in the ND, HSFD, and YS-H groups are colored in gray, green, and blue, respectively. Circle sizes indicate the log-transformed relative abundance of each taxon. **(C, D)** Heatmaps of differentially abundant taxa at the genus **(C)** and species **(D)** levels, constructed based on normalized relative abundance data. Each row represents an individual taxon, and each column represents a sample. Color intensity corresponds to scaled abundance. LDA, linear discriminant analysis; LEfSe, linear discriminant analysis effect size; ND, normal diet; HSFD, high-sugar high-fat diet; YS-H, high-dose C. sativum extract.

To characterize group-specific microbial differences at higher taxonomic resolution, linear discriminant analysis effect size (LEfSe) was applied to identify discriminant taxa among the ND, HSFD, and *Coriandrum sativum* seed extract high-dose (2.0 g/kg) groups. The analysis was conducted at both the genus and species levels and further supported by heatmap visualization (**Figures 8A–D**).

At the genus level, ND-fed mice exhibited consistent enrichment of Bacteroidota-affiliated taxa, including *Bacteroides*, *Prevotellaceae*_UCG-001, *Mucispirillum*, *Alloprevotella*, and *Monoglobus*. These genera displayed LDA scores ranging from 3.3 to 3.9, and their relative abundance was elevated in ND samples on the genus-level heatmap. Cladogram mapping showed a coherent phylogenetic distribution within Bacteroidota branches. In contrast, the HSFD group was dominated by Muribaculaceae, Dubosiella, Helicobacter, and Ileibacterium, each with LDA scores exceeding and p-values < 0.01. These genera were distributed across diverse phyla, including Proteobacteria and Actinobacteriota, and corresponded to elevated signal intensity in HSFD samples. The YS-H group demonstrated a distinct genus-level profile enriched in Firmicutes-related taxa, such as *Lactobacillus* (LDA = 4.51), *Desulfovibrio* (LDA = 4.44), *Blautia, Lachnospiraceae*_UCG-006, and *Enterorhabdus*. These genera were largely absent in the other two groups and were located primarily within Firmicutes branches in the phylogenetic tree.

Species-level analysis further refined these group-specific differences. ND-fed mice were enriched in *Bacteroides acidifaciens* (LDA = 3.94), *Mucispirillum* sp. (LDA = 3.64), and *Lactobacillus hominis* (LDA = 3.21), each displaying localized signal in ND samples and minimal representation elsewhere. HSFD mice showed increased abundance of *Helicobacter ganmani* (LDA = 4.09), *Corynebacterium stationis* (LDA = 4.42), and *Ileibacterium valens* (LDA = 3.98), reflecting the taxonomic expansion observed under high-fat dietary conditions. In the YS-H group, *Lactobacillus murinus* emerged as the most dominant discriminative species, with the highest LDA score observed (4.51, p = 0.004). Additional extract-enriched taxa included *Adlercreutzia caecicola* and *Streptococcus danieliae*, both detected exclusively in the extract-treated group with strong signal intensity and Firmicutes-based phylogenetic placement.

Genus- and species-level abundance heatmaps confirmed these patterns, showing group-specific clustering and minimal overlap among differential taxa. Several Firmicutes-affiliated taxa, including *Lactobacillus murinus*, *Lachnospiraceae*_UCG-006, and *Streptococcus danieliae*, displayed elevated relative abundance exclusively in the YS-H group, with minimal detection in the ND and HSFD samples. These taxa were consistently identified across LDA rankings, heatmap clusters, and Firmicutes-associated branches in the phylogenetic tree.

### 3.9 Correlation between extract-responsive taxa and metabolic phenotypes

**Figure 9:**
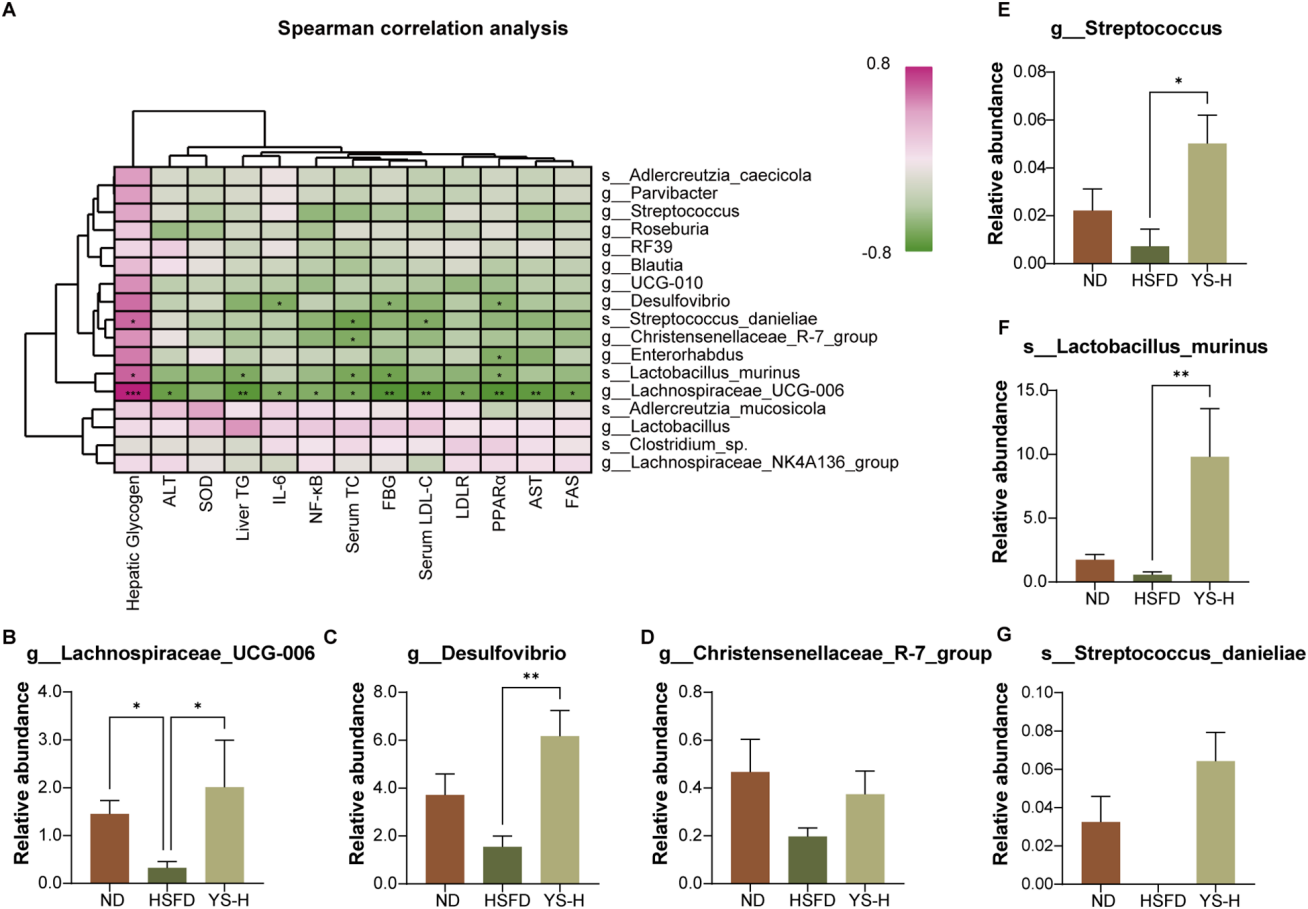
Correlation between representative microbial taxa and host metabolic parameters, and their relative abundance following *Coriandrum sativum* seed extract intervention. **(A)** Spearman correlation heatmap showing associations between differential microbial genera/species and key metabolic markers, including hepatic lipid accumulation, oxidative stress, inflammatory cytokines, and gene expression profiles. Color gradient indicates Spearman’s ρ coefficient; *p < 0.05, **p < 0.01, ***p < 0.001. **(B–G)** Relative abundance of selected bacterial taxa altered by HSFD and reversed by YS-H treatment, including **(B)** g:*Lachnospiraceae*_UCG-006, **(C)** g:*Desulfovibrio*, **(D)** g:*Christensenellaceae*_R-7_group, **(E)** g:*Streptococcus*, **(F)** s:*Lactobacillus_murinus* and **(G)** s:*Streptococcus_danieliae*. ND: normal diet; HSFD: high sugar–high fat diet; YS-H: C. sativum seed extract at 2.0 g/kg. Data are shown as mean ± SEM (n = 6); significance was assessed using one-way ANOVA followed by *post hoc* analysis.

To clarify the associations between extract-modulated gut microbiota and host metabolic outcomes, Spearman correlation analysis was performed, focusing on representative genera and species altered by high-dose *Coriandrum sativum* seed extract (**Figure 9A**). The correlation matrix incorporated indicators of glucose metabolism, lipid metabolism, liver injury, oxidative stress, and inflammation, and statistical significance was denoted using p-value thresholds (*p* < 0.05, *p* < 0.01, *p* < 0.001).

Several extract-enriched taxa demonstrated significant inverse correlations with pathological parameters induced by a high-sugar, high-fat diet. *Lachnospiraceae*_UCG-006 and *Lactobacillus murinus* showed negative correlations with fasting blood glucose, serum LDL-C, IL-6, and NF-κB levels, while positively correlating with hepatic glycogen and SOD activity. These patterns aligned with the metabolic improvements observed in extract-treated animals. *Streptococcus danieliae* and *Christensenellaceae*_R-7_group, also enriched in the extract group, exhibited inverse associations with liver injury markers (ALT, AST), lipogenesis-related genes (FAS), and systemic inflammatory indicators.

In contrast, *Desulfovibrio*, which was elevated in HSFD-fed mice, showed significant positive correlations with ALT, liver TG, and pro-inflammatory markers, while displaying negative associations with SOD and hepatic glycogen content, reflecting its alignment with a metabolically impaired state.

The relative abundance profiles of the key genera and species (**Figures 9B–G**) confirmed their differential expression across groups. *Lachnospiraceae*_UCG-006 and *Lactobacillus murinus* were markedly increased in the high-dose extract group (p < 0.05 or p < 0.01), while *Desulfovibrio* was selectively elevated in the HSFD group. *Streptococcus danieliae* was minimally detected in ND and HSFD mice but was enriched in the extract-treated group.

These data provide a taxonomic framework for understanding how specific microbial populations may contribute to the metabolic benefits observed following *Coriandrum sativum* seed extract intervention, serving as a bridge to further mechanistic investigations.

### 3.10 Functional prediction of gut microbiota via PICRUSt2

**Figure 10:**
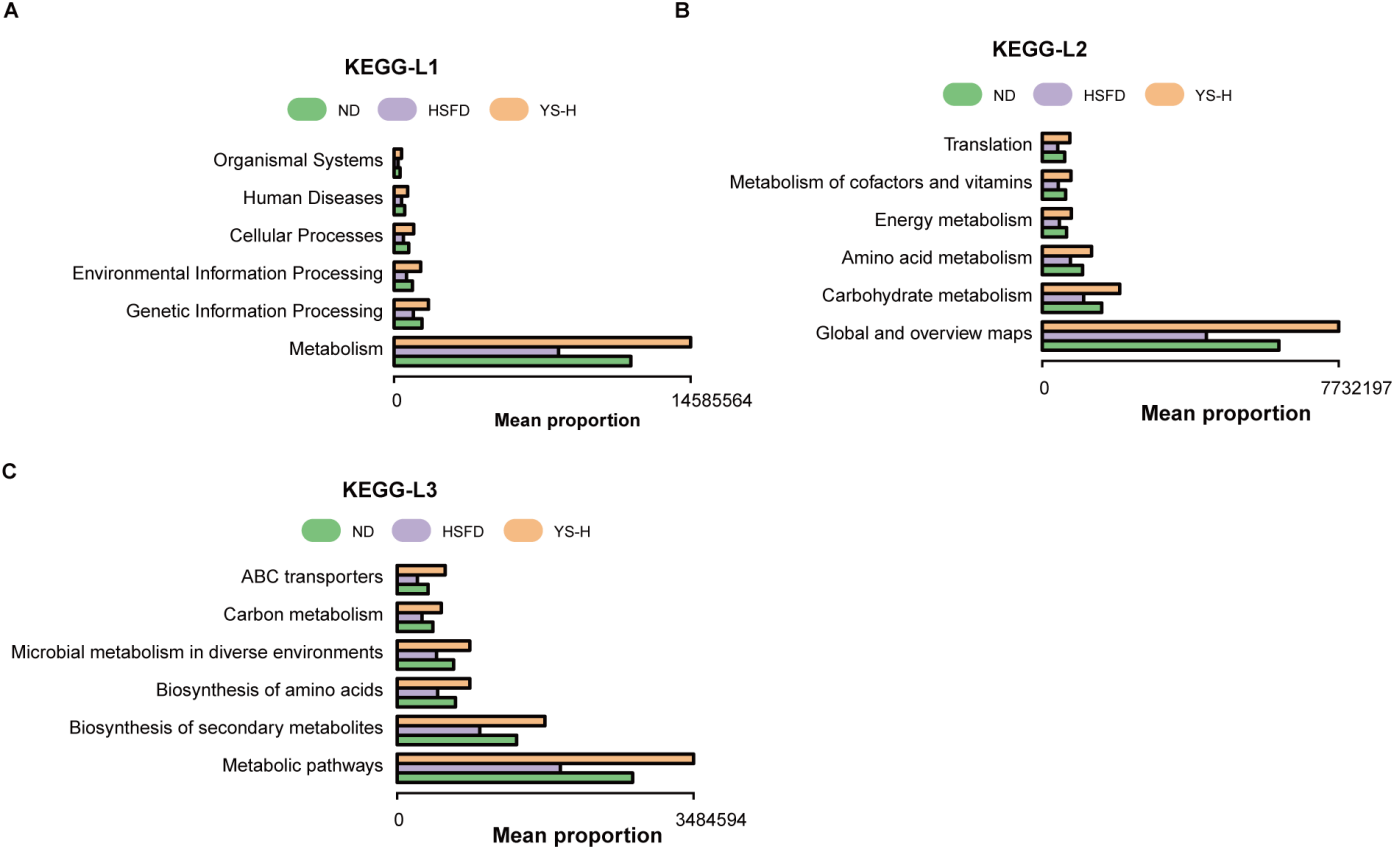
Functional prediction of gut microbiota using PICRUSt2 based on KEGG pathway enrichment analysis. **(A–C)** KEGG ortholog functional categories at levels 1 (L1), 2 (L2), and 3 (L3), respectively. The mean relative abundances of predicted microbial functions are shown across groups: **(A)** L1–major biological domains including Metabolism, Genetic Information Processing, and Environmental Information Processing. **(B)** L2–subcategories such as carbohydrate metabolism, amino acid metabolism, and energy metabolism. **(C)** L3–detailed functional modules including ABC transporters, carbon metabolism, and biosynthesis of secondary metabolites. ND: normal diet; HSFD: high sugar–high fat diet; YS-H: *Coriandrum sativum* seed extract at 2.0 g/kg.

To further investigate the functional implications of *Coriandrum sativum* seed extract-mediated microbial shifts, PICRUSt2-based predictive metagenomic profiling was performed using 16S rRNA sequencing data from the high-dose group. KEGG pathway annotations at three hierarchical levels (L1–L3) were used to assess the impact of microbial changes on host-relevant metabolic capacities (**Figures 10A–C**).

At KEGG level 1 (L1), the dominant functional category across all groups was “Metabolism”, which accounted for the largest proportion of predicted gene content. HSFD feeding modestly suppressed overall metabolic functions, while *Coriandrum sativum* extract treatment restored and further enhanced this functional category (**Figure 10A**). Other KEGG L1 pathways, such as “Genetic Information Processing” and “Environmental Information Processing”, also showed mild increases in the extract group compared to the HSFD group.

More detailed categorization at KEGG level 2 (L2) revealed extract-induced enhancements in microbial pathways related to “Carbohydrate metabolism”, “Amino acid metabolism”, and “Energy metabolism” (**Figure 10B**). Notably, the abundance of genes associated with carbohydrate metabolism was substantially increased in the *Coriandrum sativum*-treated group relative to both ND and HSFD groups, indicating a restoration of fermentative capacity that may contribute to improved glycemic control. Similarly, pathways associated with amino acid turnover and cofactor/vitamin metabolism were elevated, suggesting enhanced microbial biosynthetic potential under extract intervention.

At KEGG level 3 (L3), further resolution identified enrichment in specific metabolic modules including “Metabolic pathways”, “Biosynthesis of secondary metabolites”, and “Microbial metabolism in diverse environments” (**Figure 10C**). Among them, “Metabolic pathways” represented the most prominent shift, showing a marked increase in mean gene abundance in the *Coriandrum sativum* extract group. Additionally, elevated prediction scores for “Biosynthesis of amino acids”, “Carbon metabolism”, and “ABC transporters” indicated a broader reprogramming of microbial functional capacity toward nutrient utilization and environmental adaptability.

Collectively, these predictions indicate that *Coriandrum sativum* seed extract potentially regulates key functions in energy production, biosynthesis, and host-microbiome interactions, as a potential basis for the improved metabolism seen in mice on a high-sucrose high-fat diet (HSFD).

## 4 Discussion

Emerging evidence highlights the pivotal role of gut microbiota and hepatic-lipid-glucose axis in mediating the beneficial effects of dietary phytochemicals on metabolic disorders (49–51). In this study, we systematically evaluated the metabolic regulatory capacity of *Coriandrum sativum* seed aqueous extract in a diet-induced mouse model of glucolipid metabolism disorder, revealing coordinated improvements in systemic metabolic markers, hepatic histopathology, and microbial ecology. The following discussion integrates our findings with existing literature to elucidate the mechanistic underpinnings and significance of the observed effects.

The phytochemical composition of *Coriandrum sativum* seed aqueous extract provided the biochemical foundation for its observed bioactivity. Consistent with previous reports, the extract was rich in polyphenols and flavonoids, including linalool, quercetin derivatives, and phenolic acids (52). These compounds have been linked to antioxidant, anti-inflammatory, and metabolic regulatory effects through mechanisms such as AMPK activation, ROS scavenging, and modulation of gut microbiota (53–56). Notably, the presence of these bioactives in an aqueous extractable form enhances their dietary applicability and potential for clinical translation. The antioxidant activities of these components may directly counteract oxidative stress and lipid peroxidation, both key features of metabolic syndrome (28, 57, 58).

Consistent with the pathophysiology of diet-induced metabolic syndrome, mice in the HSFD group showed significant increases in body weight, serum TG, TC, LDL-C, and hepatic lipid accumulation, accompanied by pronounced steatosis in histological sections. These changes were attenuated in a dose-dependent manner by *Coriandrum sativum* seed extract. The lipid-lowering effects may be partially attributed to polyphenol-mediated regulation of hepatic lipid metabolism via NF-κB signaling (59). Additionally, quercetin and its glycosides have been shown to modulate PPARα expression, enhancing β-oxidation and reducing hepatic TG content (60). These findings align with the improved liver morphology observed in histological analysis, highlighting a hepatoprotective effect likely driven by restored lipid homeostasis and suppressed lipogenesis.

The HSFD group exhibited elevated fasting glucose and insulin levels, along with impaired HOMA-IR and β-cell function, consistent with insulin resistance and early pancreatic stress. Treatment with *Coriandrum sativum* extract improved glycemic indices and preserved pancreatic architecture. These effects align with previous reports showing that *Coriandrum sativum* components enhance insulin sensitivity (59). Flavonoids such as rutin and apigenin also enhance β-cell survival by attenuating ER stress and reducing oxidative damage (61, 62). Beyond direct modulation of glycemic signaling, the extract’s attenuation of systemic inflammation and lipid overload may secondarily relieve β-cell burden, contributing to preserved islet morphology and endocrine function (34).

The gut microbiota has been increasingly recognized as a key integrative hub linking dietary inputs to host metabolic outcomes (37). In this context, our findings suggest that the metabolic benefits of *Coriandrum sativum* seed extract are at least partly mediated through its capacity to remodel gut microbial composition. The pronounced enrichment of *Faecalibaculum* in the HSFD group is notable, as this genus has been linked to intestinal barrier disruption and increased IL-6 secretion in metabolic inflammation models (63, 64). Parallel elevation of Desulfobacterota, is consistent with its role in hydrogen sulfide overproduction, which can compromise tight junctions (65, 66).

*Lachnospiraceae*_NK4A136_group also re-emerged in the extract group, albeit variably. This taxon comprises butyrate-producing strains, which have demonstrated regulatory effects on Treg differentiation, histone deacetylase inhibition, and maintenance of mucosal integrity in metabolic disease contexts (67–69). Given its depletion under HSFD exposure, the detectable rebound of this genus supports a potential immunometabolic benefit associated with the intervention.

While *Lactobacillus* abundance remained low overall, its reappearance in certain extract-treated samples is still relevant. Species within this genus have been implicated in bile salt hydrolase activity, modulation of FXR and TGR5 pathways, and attenuation of dyslipidemia and insulin resistance in diet-induced models (70, 71). Even moderate recovery may contribute to local metabolic tuning within the intestinal lumen.

Genera such as *Eubacterium_coprostanoligenes*_group, *Dubosiella*, and *Allobaculum* fluctuated inconsistently across groups, with no clear directional shift attributable to the intervention (72, 73). These taxa have been previously reported in both homeostatic and pathologic contexts, underscoring the need for species-level resolution and functional readouts before assigning mechanistic significance.

The genus-level profile in *Coriandrum sativum*-treated mice thus reflects a partial realignment toward health-associated taxa, particularly those involved in SCFAs (short-chain fatty acids) production and carbohydrate metabolism. However, the incomplete suppression of harmful genera and pronounced inter-sample heterogeneity indicate that the intervention modulates, but does not normalize, HSFD-induced dysbiosis. These shifts warrant further validation through metabolomic and barrier integrity assays to clarify their relevance to host metabolic outcomes.

The LEfSe-based taxonomic analysis further deepens our understanding of the extract’s microbiota-mediated effects by highlighting specific microbial signatures associated with metabolic benefit. Notably, *Lactobacillus murinus* emerged as a top biomarker in the YS-H group, aligning with prior reports of its anti-inflammatory and antioxidative potential via gut barrier reinforcement and immune regulation (74, 75). Similarly, the increase in *Lachnospiraceae*_UCG-006 is particularly compelling, given its butyrate-producing capacity and prior links to improved insulin sensitivity, intestinal barrier integrity, and lipid homeostasis (76–78). These taxa are not only functionally active in SCFA metabolism but also capable of modulating bile acid signaling and systemic cytokine responses (79–81). Thus, beyond compositional restoration, the extract appears to select for functionally specialized microbes with established roles in energy regulation and hepatic protection. Such selective enrichment reinforces the concept of “precision prebiotic” effects, where plant-derived polyphenols shape microbial ecology toward defined metabolic outcomes.

To bridge microbial shifts with host phenotypes, correlation analysis revealed a coherent axis linking beneficial taxa with metabolic improvements. Genera such as *L. murinus*, *Lachnospiraceae*_UCG-006, and *S. danieliae* displayed robust negative associations with hepatic lipid content, serum LDL-C, inflammatory cytokines, and liver enzymes, while positively correlating with hepatic glycogen and antioxidant status. These associations lend credence to the hypothesis that microbial restoration is not merely a consequence but an active mediator of the observed metabolic benefits. Similar correlations have been reported in studies where targeted enrichment of butyrate-producers alleviated NAFLD and systemic insulin resistance through epigenetic modulation of hepatic gene expression (82, 83). Together, these data suggest that *Coriandrum sativum* extract exerts systemic benefits not solely through direct hepatic modulation, but via microbiota-dependent signaling networks involving SCFAs, bile acids, and immunometabolic crosstalk.

Our findings suggest that *Coriandrum sativum* seed aqueous extract exerts coordinated metabolic benefits through hepatic transcriptional regulation, inflammatory inhibition, oxidative stress attenuation, and gut microbiota modulation in a diet-induced murine model. These effects were observed in a dose-dependent manner and supported by both phenotypic and molecular evidence. Nonetheless, further work is warranted to isolate and validate the active compounds responsible for these effects, and to explore the upstream signaling pathways that mediate the observed hepatic and intestinal responses. In addition, future studies may benefit from applying metabolomics or germ-free animal models to more precisely define the role of microbiota in shaping host metabolic outcomes. These directions build upon the current findings and may help to better characterize the therapeutic potential and translational value of *Coriandrum sativum* as a functional dietary component in the context of metabolic disorders.

## 5 Conclusion

The present study provides pharmacological evidence that *Coriandrum sativum* seed aqueous extract exerts regulatory effects on glucolipid metabolic disturbances induced by high-fat and high-sugar dietary intake. The extract markedly improved systemic lipid and glucose parameters, attenuated hepatic steatosis, and preserved pancreatic and colonic tissue integrity. These therapeutic effects were mechanistically associated with transcriptional modulation of hepatic lipid oxidation and inflammatory pathways, enhancement of antioxidant defense, and selective remodeling of gut microbial ecology. The enrichment of SCFAs–producing taxa and suppression of endotoxin-generating bacteria further reflect its microbiota-mediated efficacy. Collectively, these findings support the potential application of *Coriandrum sativum* seed as a multi-target dietary intervention in the integrative management of metabolic disorders.

## Data Availability

The original contributions presented in the study are included in the article/**Supplementary Material**. Further inquiries can be directed to the corresponding authors.
