# Supplementary material for: Protective role of aqueous Coriandrum sativum seed extract in diet-induced glucolipid metabolic disorder through gut–liver axis regulation
Source: Front Endocrinol (Lausanne). 2026 Feb 13;17:1744741. doi: 10.3389/fendo.2026.1744741 (PMC12946750; doi:10.3389/fendo.2026.1744741)
Supplement: Supplementary file 1 [file DataSheet1.docx]

Supplementary Material

# Supplementary Tables

**Table 1.** GC-MS analytical parameters of identified compounds in C. sativum seed ex-tract

| Number | compounds | Retention time(min) | Retention Index | Formula |
| --- | --- | --- | --- | --- |
| 1 | 2,6-dimethylocta-3,7-diene-2,6-diol | 14.837 | 1197 | C_10_H_18_O_2_ |
| 2 | 4-ethenyl-2-methoxyphenol | 21.522 | 1293 | C_9_H_10_O_2_ |
| 3 | 3-methylhepta-1,6-dien-3-ol | 23.453 | 888 | C_8_H_14_O |
| 4 | (2E)-2,6-dimethylocta-2,7-diene-1,6-diol | 24.923 | 1325 | C_10_H_18_O_2_ |
| 5 | Dodecanal | 27.528 | 1402 | C_12_H_24_O |
| 6 | Hexadecane | 31.952 | 1612 | C_16_H_34_ |
| 7 | 2,6,10,14-tetramethylhexadecane | 49.709 | 1753 | C_20_H_42_ |
| 8 | Hexadecanoic acid | 58.223 | 1968 | C_16_H_32_O_2_ |
| 9 | Octadecanoic acid | 66.304 | 2167 | C_18_H_36_O_2_ |

The retention time, retention index, and molecular formula of compounds in C. sa-tivum seed aqueous extract were determined by gas chromatography–mass spectrom-etry (GC-MS). All compounds were identified based on comparison with standard mass spectral libraries. No internal standards were applied. Abbreviations: GC-MS, gas chromatography–mass spectrometry.

**Table 2.** Spectral matching and relative abundance of characterized compounds in C. sativum seed extract

| Number | Compounds | CAS | SI | Conc./% |
| --- | --- | --- | --- | --- |
| 1 | 2,6-dimethylocta-3,7-diene-2,6-diol | 13741-21-4 | 93 | 1.35 |
| 2 | 4-ethenyl-2-methoxyphenol | 7786-61-0 | 94 | 11.62 |
| 3 | 3-methylhepta-1,6-dien-3-ol | 34780-69-3 | 90 | 1.08 |
| 4 | (2E)-2,6-dimethylocta-2,7-diene-1,6-diol | 64142-78-5 | 94 | 2.58 |
| 5 | Dodecanal | 112-54-9 | 95 | 0.51 |
| 6 | Hexadecane | 544-76-3 | 94 | 1.1 |
| 7 | 2,6,10,14-tetramethylhexadecane | 638-36-8 | 90 | 1.47 |
| 8 | Hexadecanoic acid | 57-10-3 | 94 | 6.13 |
| 9 | Octadecanoic acid | 57-11-4 | 91 | 4.28 |

**Supplementary Table A1.** Primer sequences used for quantitative RT-PCR analysis.

| Gene | Forward Primer (5′→3′) | Reverse Primer (5′→3′) |
| --- | --- | --- |
| PPARα | GGGCAAGAGAATCCACGAAG | GTTGTTGCTGGTCTTTCCCG |
| FAS | TGGTGGTGTGGACATGGTCACAGA | CCGAAGCTGGGGGTCCATTGTGTG |
| LDLR | CATCCTCGGACATCCACCC | TTCGGTCGTGGCACAAGAAC |
| NF-κB | GGCCTCATCCACATGAACTT | CACTGTCACCTGGAAGCAGA |
| IL-6 | CAAAGCCAGAGTCCTTCAGAG | AGCATTGGAAATTGGGGTAG |
| β-actin | GGCTGTATTCCCCTCCATCG | CCAGTTGGTAACAATGCCATGT |

Forward and reverse primer sequences (5′→3′) used for quantitative real-time polymerase chain reaction (RT-PCR) analysis of gene expression. Abbreviations: PPARα, peroxisome proliferator-activated receptor alpha; FAS, fatty acid synthase; LDLR, low-density lipoprotein receptor; NF-κB, nuclear factor kappa-B; IL-6, interleukin-6; β-actin, beta-actin (reference gene); RT-PCR, quantitative real-time polymerase chain reaction.

**Supplementary Table A2.** Representative Mass Spectral Fragmentation Patterns of Compounds Identified in C. sativum Seed Aqueous Extract

| Number | Compounds | m/z | Absolute Intensity |
| --- | --- | --- | --- |
|  | 2,6-dimethylocta-3,7-diene-2,6-diol | 43.05 | 35077 |
|  |  | 67.05 | 26330 |
|  |  | 82.05 | 48494 |
| 1 |  | 91.15 | 992 |
|  |  | 109 | 989 |
|  |  | 125.1 | 726 |
|  |  | 137.1 | 354 |
|  | 4-ethenyl-2-methoxyphenol | 51 | 42452 |
|  |  | 63 | 24580 |
|  |  | 77.05 | 125920 |
| 2 |  | 89.05 | 17629 |
|  |  | 107.05 | 111196 |
|  |  | 150.05 | 290497 |
|  |  | 151.05 | 27868 |
|  | 3-methylhepta-1,6-dien-3-ol | 43.05 | 19529 |
|  |  | 55.05 | 10537 |
| 3 |  | 71.05 | 52119 |
|  |  | 93.05 | 1299 |
|  |  | 111 | 1191 |
|  | (2E)-2,6-dimethylocta-2,7-diene-1,6-diol | 43.05 | 37008 |
|  |  | 67.05 | 21775 |
|  |  | 71.05 | 21765 |
| 4 |  | 93.1 | 4323 |
|  |  | 110.1 | 2709 |
|  |  | 119.1 | 2793 |
|  |  | 137.1 | 1941 |
|  |  | 152 | 282 |
|  |  | 43.05 | 10211 |
|  | Dodecanal | 57.05 | 10824 |
|  |  | 82.1 | 5528 |
|  |  | 96.1 | 2754 |
| 5 |  | 109.1 | 851 |
|  |  | 123.05 | 484 |
|  |  | 140.1 | 784 |
|  |  | 156.2 | 164 |
|  | hexadecane | 43.05 | 40372 |
|  |  | 57.05 | 43426 |
|  |  | 71.1 | 38892 |
|  |  | 99.05 | 6972 |
|  |  | 113.1 | 5657 |
|  |  | 127.15 | 3174 |
| Number | Compounds | m/z | Absolute Intensity |
| 6 |  | 141.05 | 884 |
|  |  | 155.1 | 1696 |
|  |  | 169.1 | 1280 |
|  |  | 183 | 142 |
|  |  | 211 | 40 |
|  | 2,6,10,14-tetramethylhexadecane | 43.05 | 9988 |
|  |  | 57.05 | 17381 |
|  |  | 71.1 | 12999 |
|  |  | 99.05 | 2313 |
|  |  | 113.2 | 1329 |
| 7 |  | 127.1 | 1122 |
|  |  | 141.05 | 484 |
|  |  | 183.1 | 262 |
|  |  | 225.2 | 15 |
|  |  | 239.2 | 86 |
|  |  | 253.2 | 77 |
|  |  | 282.1 | 68 |
|  | hexadecanoic acid | 43.05 | 32852 |
|  |  | 59.95 | 23307 |
|  |  | 73 | 26633 |
|  |  | 97.1 | 5670 |
|  |  | 115 | 3279 |
|  |  | 129.1 | 8440 |
| 8 |  | 143.05 | 1943 |
|  |  | 157.1 | 2493 |
|  |  | 171.1 | 2393 |
|  |  | 185.1 | 1857 |
|  |  | 213.15 | 2284 |
|  |  | 227.05 | 536 |
|  |  | 239.2 | 107 |
|  |  | 256.15 | 2695 |
|  | octadecanoic acid | 43.05 | 39637 |
|  |  | 60 | 22641 |
|  |  | 73 | 25908 |
|  |  | 97.05 | 6129 |
|  |  | 115.05 | 2727 |
|  |  | 129 | 9252 |
|  |  | 143.05 | 1395 |
| 9 |  | 157.1 | 1057 |
|  |  | 171.1 | 1223 |
|  |  | 185.15 | 2545 |
|  |  | 199.05 | 1117 |
|  |  | 227.2 | 722 |
| Number | Compounds | m/z | Absolute Intensity |
|  |  | 241.15 | 1971 |
|  |  | 255.3 | 307 |
|  |  | 284.2 | 3506 |

lute signal intensities were recorded for the major fragment ions of compounds identified by GC-MS. Data represent characteristic fragmentation patterns derived from total ion chromatograms. Abbreviations: m/z, mass-to-charge ratio; GC-MS, gas chromatography–mass spectrometry.

# Supplementary Figures


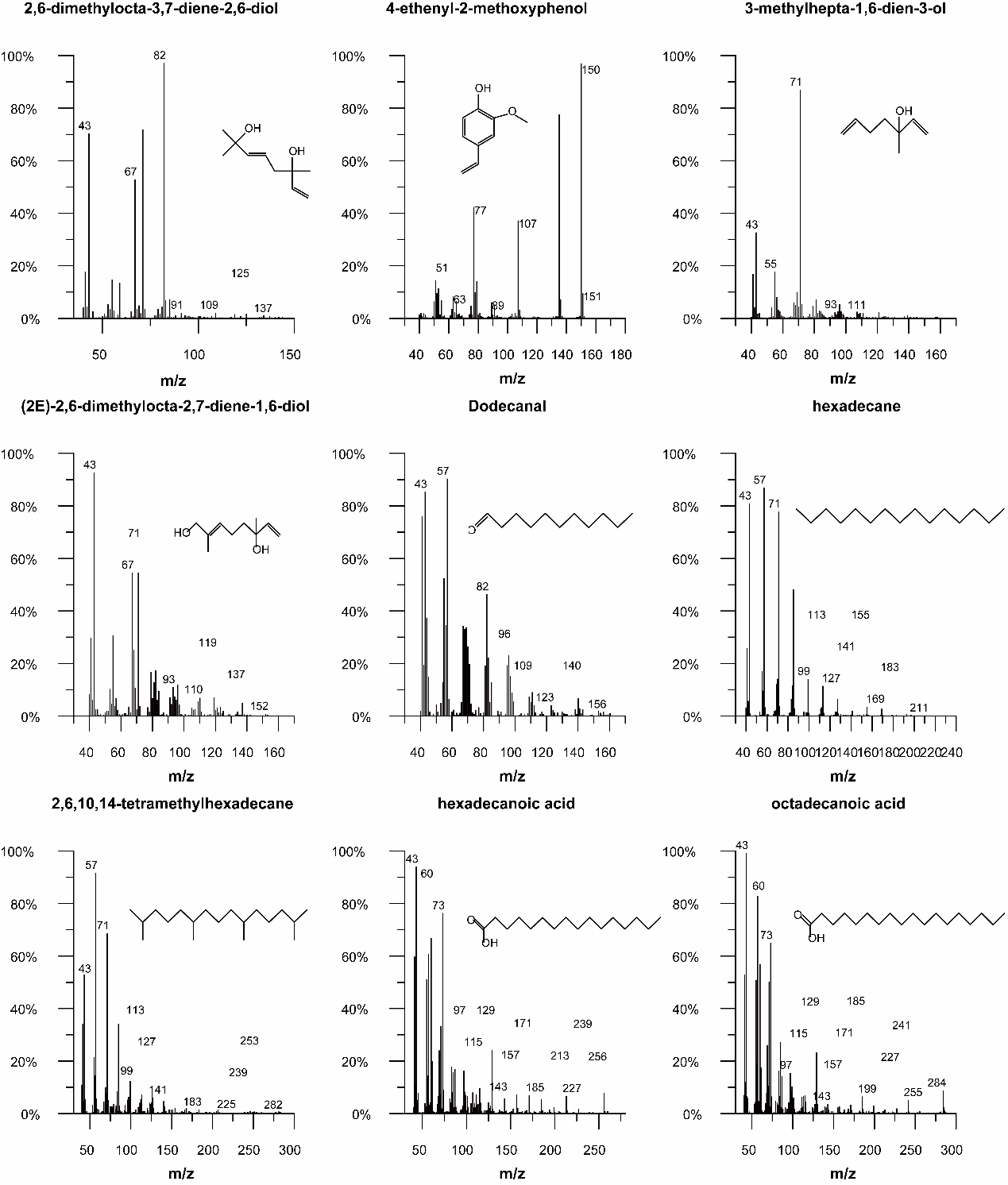


**Supplementary Figure A1.** Representative electron ionization mass spectra of compounds identified in aqueous extract of Coriandrum sativum seed.

Typical electron ionization (EI) mass spectra of nine major constituents detected in the aqueous extract of Coriandrum sativum seed by gas chromatography–mass spectrometry (GC-MS). The compounds include: 2,6-dimethylocta-3,7-diene-2,6-diol, 4-ethenyl-2-methoxyphenol, 3-methylhepta-1,6-dien-3-ol, (2E)-2,6-dimethylocta-2,7-diene-1,6-diol, dodecanal, hexadecane, 2,6,10,14-tetramethylhexadecane, hexadecanoic acid, and octadecanoic acid. Fragment ions are annotated with their corresponding mass-to-charge ratios (m/z). Each panel presents the relative intensity (%) of characteristic fragment ions and the chemical structure of the corresponding compound. Abbreviations: EI, electron ionization; GC-MS, gas chromatography–mass spectrometry; m/z, mass-to-charge ratio.


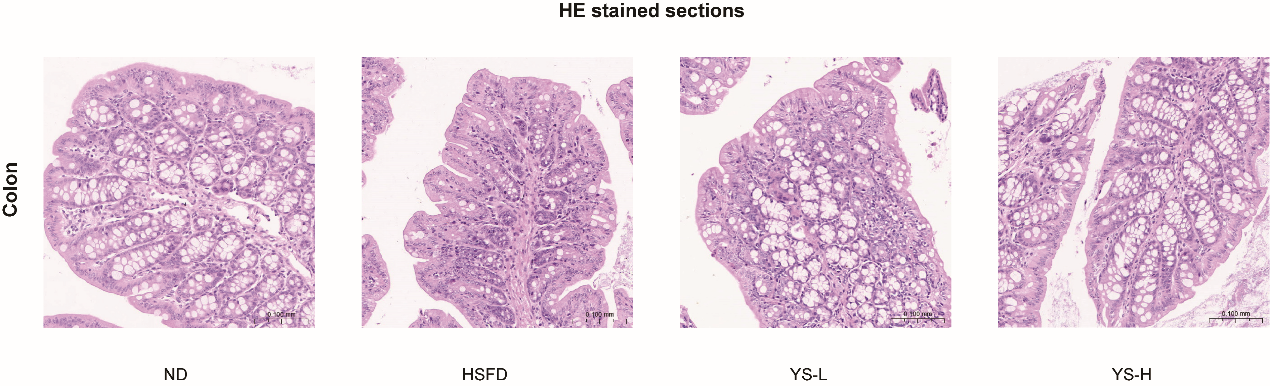


**Supplementary Figure A2.** Histopathological changes in colonic tissues assessed by hematoxylin and eosin (HE) staining

Representative HE-stained sections of the colon from each experimental group. Mice fed a high-sugar high-fat diet (HSFD) exhibited marked histological abnormalities, including epithelial disorganization, crypt distortion, and mucosal barrier disruption, compared with the normal diet (ND) group. Administration of aqueous Coriandrum sativum seed extract, particularly at the high dose (YS-H, 2.0 g/kg), ameliorated these pathological alterations, showing improved crypt architecture and preserved mucosal structure. YS-L: low dose extract (1.0 g/kg). Scale bar = 100 μm.
